# Lipid Nanoparticles With Fine‐Tuned Composition Show Enhanced Colon Targeting as a Platform for mRNA Therapeutics

**DOI:** 10.1002/advs.202408744

**Published:** 2024-11-25

**Authors:** Riccardo Rampado, Gonna Somu Naidu, Olga Karpov, Meir Goldsmith, Preeti Sharma, Assaf Ezra, Lior Stotsky, Dor Breier, Dan Peer

**Affiliations:** ^1^ Laboratory of Precision Nanomedicine Shmunis School of Biomedicine and Cancer Research Tel Aviv University Tel Aviv‐Yafo 69978 Israel; ^2^ Department of Materials Sciences and Engineering Tel Aviv University Tel Aviv‐Yafo 69978 Israel; ^3^ Center for Nanoscience and Nanotechnology Tel Aviv University Tel Aviv‐Yafo 69978 Israel; ^4^ Cancer Biology Research Center Tel Aviv University Tel Aviv‐Yafo 69978 Israel

**Keywords:** colon, genetic medicines, inflammatory bowel disease, lipid nanoparticles, mRNA, targeting

## Abstract

Lipid Nanoparticles (LNPs) recently emerged as an invaluable RNA delivery platform. With many LNP‐based therapeutics in the pre‐clinical and clinical pipelines, there is extensive research dedicated to improving LNPs. These efforts focus mainly on the tolerability and transfectability of new ionizable lipids and RNAs, or modulating LNPs biodistribution with active targeting strategies. However, most formulations follow the well‐established lipid proportions used in clinically approved products. Nevertheless, investigating the effects of LNPs composition on their biodistribution can expand the toolbox for particle design, leading to improved delivery strategies. Herein, a new LNPs (30‐n‐LNPs) formulation with increasing amounts of phospholipids is investigated as a possible mRNA delivery system for treating Inflammatory Bowel Diseases. Compared to LNPs with benchmark composition (b‐LNPs), n‐LNPs containing 30% distearoylphosphatidylcholine (DSPC) are well tolerated following intravenous administration and display natural targeting toward the inflamed colon in dextran sodium sulfate (DSS)‐colitis bearing mice, while de‐targeting clearing organs such as the liver and spleen. Using interleukin‐10‐encoding mRNA as therapeutic cargo, n‐LNPs demonstrated a reduction of pathological burden in colitis‐bearing mice. n‐LNPs represent a starting point to further investigate the influence of LNPs composition on systemic biodistribution, ultimately opening new therapeutic modalities in different pathologies.

## Graphical abstract

This work investigates the influence of increasing phospholipids on lipid nanoparticles (LNPs) biodistribution. 30‐n‐LNPs with a new composition are proposed, displaying colon targeting and lower liver and spleen‐mediated clearance compared to benchmark LNPs. This tropism appears mediated by myeloid cells, and the therapeutic potential of IL‐10 mRNA‐loaded 30n‐LNPs is demonstrated in the inflamed colon, reducing inflammatory cytokines levels and tissue damage.

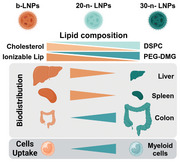

## 1 Introduction

Inflammatory Bowel Diseases (IBDs) are chronic inflammatory pathologies of the gastrointestinal tract that affect ≈7 million people worldwide.^[^ ^1^ ^]^ Despite the extensive research on this topic, no single clear pathogenic mechanism for IBDs has been elucidated. The main culprits include genetic predisposition, immunological dysfunctions, gut microbiota alteration, but also environmental factors.^[^ ^2^ ^]^ IBDs are normally classified as either Crohn's Disease or Ulcerative Colitis, based on their localization in the gastrointestinal tract and the depth of tissue inflammation and damage.^[^ ^3^, ^4^ ^]^ IBDs symptoms generally include intestinal bleeding, long‐term fibrosis, abdominal pain, and diarrhea.^[^ ^5^ ^]^ IBDs are also considered a major risk for the development of colorectal cancer.^[^ ^6^ ^]^ The current treatments for IBDs are still largely based on traditional anti‐inflammatory and immune‐suppressive agents, and more recently on monoclonal antibodies and small molecules that modulate immune signaling pathways.^[^ ^7^, ^8^ ^]^ Unfortunately, none of these approaches are curative and they often lose efficacy over time. These circumstances have a very negative impact on the patient's quality of life, causing chronic discomfort, and requiring constant medication in clinical settings.

Among the many cytokines involved in IBDs, interleukin 10 (IL‐10) gathered a lot of attention since dysfunction of its signaling is considered a major contributor to the development of IBDs.^[^ ^8^ ^]^ IL‐10 can reduce intestinal inflammation by downregulating pro‐inflammatory cells and promoting the differentiation of immunosuppressive T_reg_ lymphocytes in the gut.^[^ ^8^, ^9^, ^10^ ^]^ The direct systemic administration of IL‐10 has shown some efficacy.^[^ ^11^ ^]^ However, this approach would require the administration of large amounts of protein, which have been related to increased IFNγ and side effects like anemia and thrombocytopenia.^[^ ^12^, ^13^, ^14^ ^]^ In this context, mRNA‐loaded lipid nanoparticles (LNPs) represent a new possible approach to re‐establish intestinal immune homeostasis and hinder IBDs’ symptoms. Indeed, LNPs can deliver IL‐10‐encoding mRNA to the inflamed intestinal tissue. The in situ production of this cytokine would address the instability of IL‐10 while avoiding systemic effects by carrying the mRNA specifically to the affected tissue.

Lipid Nanoparticles (LNPs) are currently among the most advanced non‐viral vectors used to deliver nucleic acids with several clinically approved indications.^[^ ^15^, ^16^, ^17^, ^18^, ^19^ ^]^ In particular, the fast development and approval of the first mRNA vaccines to fight COVID‐19 significantly boosted the relevance of LNPs and put these drug delivery systems (DDSs) in the spotlight. Most of the current research is focused on exploring new mRNA cargo designs to improve its stability, lower its immunogenicity, and increase its transfection efficiency.^[^ ^20^ ^]^ Other lines of inquiry focus on synthesizing and testing new lipids, especially new ionizable lipids, which are the major contributors to LNPs‐mediated mRNA transfection.^[^ ^16^ ^]^ Many studies follow a more traditional approach, developing LNPs formulations functionalized with a variety of active targeting moieties, to improve their accumulation in the desired tissues.^[^ ^21^ ^]^ However, the success of the COVID‐19 vaccines somewhat cemented what is the ideal LNP lipid molar proportion ratio (50% ionizable lipid, 38.5% cholesterol, 10% of helper lipid, and 1.5% PEGylated lipid), making this the “benchmark” LNPs composition.^[^ ^22^ ^]^ Nevertheless, there are ongoing efforts that aim to explore new LNPs compositions and their biological repercussions on major organs such as the lungs, the spleen, and the liver.^[^ ^23^, ^24^, ^25^, ^26^, ^27^, ^28^, ^29^, ^30^, ^31^, ^32^ ^]^ In particular, a recent work from Chander et al demonstrated how the higher percentages of helper lipids could improve mRNA expression.^[^ ^33^ ^]^

**Figure 1:**
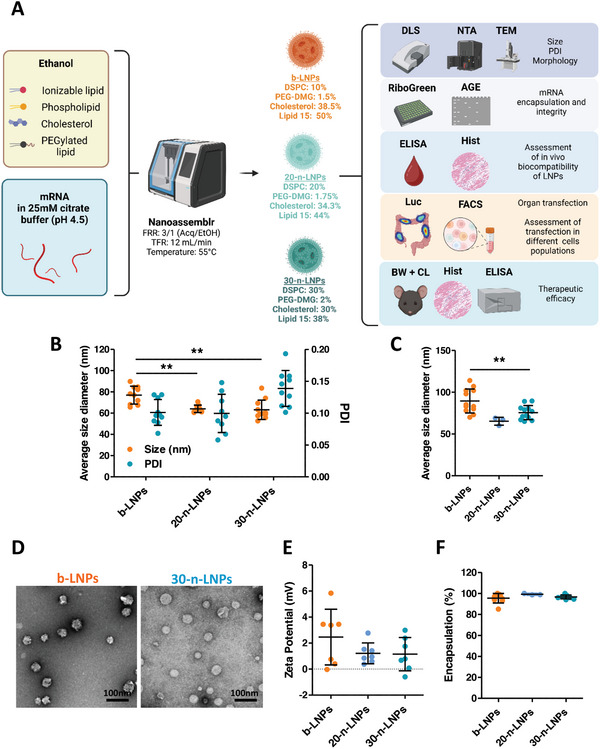
Physicochemical and structural characterization of LNPs. A) Schematic representation of the methods used for LNPs formulation and their characterization (AGE: agarose gel electrophoresis; Luc: in vivo Luc expression; FACS: Flow Cytometry; BW: body weight assessment; CL: colon length; Hist: histology). B) Summarized size and PDI of b‐LNPs and n‐LNPs measured by DLS (*n* = 9). C) LNPs size measured by NTA (*n* = 12). D) Measurement of particle diameter performed on TEM images (Data presented as Box‐Whiskers from minimum to maximum), exemplified in (E). F) zeta potential measurement (*n* = 6) and Ribogreen mRNA encapsulation assessment (*n* = 7, G). All data are presented as average ± SEM; Statistical analysis was performed using a one‐way ANOVA test (^*^ *p* < 0.05, ^**^ *p* < 0.01).

Following these observations, in this study, we developed and tested a new LNPs DDS characterized by the increased molar percentages of the helper lipid DSPC (20% and 30%, termed 20‐n‐LNPs and 30‐n‐LNPs, respectively) compared to the current benchmark formulation represented by the COVID‐19 vaccines composition (10% DSPC, b‐LNPs **Figure** **1**A). We performed a direct comparison between these new LNPs formulations, including physical characterization, in vitro testing, and in vivo assessment of their biodistribution. We demonstrated how increased DSPC levels resulted in significant improvement of colonic mRNA expression and decreased liver and spleen off‐target accumulation in healthy and colitis‐bearing mice. Finally, after selecting the LNPs with 30% DSPC as our leading formulation, we tested their viability as therapeutic DDS by loading them with an IL‐10‐encoding mRNA, confirming their efficacy in a murine model of IBDs.

## 2 Results and Discussion

### 2.1 Characterization of LNPs

LNPs were formulated as described in the methods section using luciferase mRNA (mLuc) as model cargo. Lipid 15 was selected as a lead ionizable lipid following the result from previous studies performed by our team (Figure S1, Supporting Information).^[^ ^34^ ^]^ Dynamic Light Scattering (DLS) measurements revealed a significant difference between the size of b‐LNPs (78.98 ± 7.98 nm), 20‐n‐LNPs (64.04 ± 1.15 nm), and 30‐n‐LNPs (64.60 ± 3.68 nm), with both formulations showing high uniformity (Figure 1B). As a secondary measurement, LNPs diameter was also measured using nanoparticle tracking analysis (NTA, Figure 1C; Figures S2A–C, Supporting Information). NTA results corroborated that the 20‐n‐LNPs and 30‐n‐LNPs are significantly smaller (65.33 ± 4.72 nm and 75.59 ± 8.45 nm respectively) than the b‐LNPs (89.6 ± 4.15 nm). Furthermore, NTA reported different concentrations between the two formulations, with 20‐n‐LNPs and 30‐n‐LNPs being more concentrated (8.70 ± 1.41·10^12^ and 1.12 ± 0.11·10^13^ particles mL^−1^) than b‐LNPs (7.30·10^12^ particles mL^−1^, Figure S2D, Supporting Information). LNPs size and shape were also assessed using transmission electron microscopy (TEM, Figure 1D) and cryo‐electron microscopy (Cryo‐EM, Figure S1E, Supporting Information) for b‐LNPs and 30‐n‐LNPs, confirming the LNPs’ spheroidal shape, with both having a dense core surrounded by an outer layer, possibly consisting of PEG chains.

All particles displayed a slightly positive surface charge without significant differences between them (ζ, Figure 1E). When measuring the ζ potential across different ranges of pH in 0.1X PBS (Figure S2F, Supporting Information), all formulations displayed the same isoelectric point (IP), estimated to be between pH 6.4 and 6.9. This proves how the LNPs IP is not dependent on the proportion of Lipid 15 used in the formulation. Furthermore, at physiological pH (7.4), both formulations were slightly negatively charged, a feature suitable for systemic administration.

Next, to test the encapsulation efficiency of the mRNA, we performed a RiboGreen assay as previously reported.^[^ ^35^, ^36^ ^]^ The analysis revealed an almost complete nucleic acid encapsulation for both LNPs formulations (b‐LNPs: 95.45 ± 4.56%; 20‐n‐LNPs: 87.77 ± 0.44%; and 30‐n‐LNPs: 96.6 ± 1.79%, respectively, Figure 1F). This was further confirmed by agarose gel electrophoresis (Figure S2G, Supporting Information).

In vitro testing of the LNPs showed that both are well tolerated in multiple cell lines (Figure S3, Supporting Information). Finally, both formulations were found to be stable when stored in 4 °C (Figures S4A–C, Supporting Information). Taken together, these results show that both b‐LNPs and n‐LNPs have similar physicochemical properties and are suitable for further in vitro and in vivo studies.

### 2.2 In Vitro mRNA Expression

Following the characterization, mLuc‐loaded LNPs were tested in vitro in RAW 264.7 cells as a macrophage cell line that can simulate the in vivo behavior of the gut macrophages. We compared stimulated (INFL) and unstimulated (UT, naïve) cells. Cellular inflammation was confirmed by assessing the change of cell morphology using fluorescence microscopy and estimating the overexpression of the M1 polarization marker CD64 using multiple methods (Figures S5A–C, Supporting Information). As displayed in Figure S5D (Supporting Information), while in general inflamed cells showed better transfection efficiency compared to naïve cells, the 20‐n‐LNPs and 30‐n‐LNPs were found to be less sensitive to the change in cellular state, a result that is highly relevant when aiming to reach a site of inflammation. While 20‐n‐LNPs had similar transfection efficiency in other cell lines compared to b‐LNPs, 30‐n‐LNPs were less effective in transfecting cells (Figure S6, Supporting Information).

Next, we tested the functionality of IL‐10 mRNA (mIL‐10) in vitro. Thus, we prepared LNPs with mIL‐10 and transfected multiple cell lines with both formulations, and measured IL‐10 expression after 72 h. Despite other cell lines showing a similar pattern to the one observed for Luc (Figures S7A–D, Supporting Information), inflamed RAW 264.7 cells displayed a relevant amount of IL‐10 expression when compared to their non‐stimulated counterpart (Figure S7E, Supporting Information, 0.84 ± 0.47pg mL^−1^ and 948.6 ± 207.3, respectively). This background expression has been reported in the literature for RAW 264.7 cells that use an inflammatory feedback loop to control their inflammatory state.^[^ ^37^, ^38^ ^]^ As for the LNPs‐treated monocytes, as expected, mIL‐10‐b‐LNPs showed high IL‐10 expression in UT cells (9568 ± 1984 pg mL^−1^), but their activity was dramatically reduced to levels of expression comparable to the background in the stimulated cells (1406 ± 190 pg mL^−1^). mIL‐10‐20‐n‐LNPs and mIL‐10‐30‐n‐LNPs showed significantly lower cytokine expression in UT RAW 264.7 cells (1394 ± 226.7 and 1232 ± 257.9 pg mL^−1^ respectively).

### 2.3 In Vivo Biodistribution in Healthy and in Colitis Induced Mice

**Figure 2:**
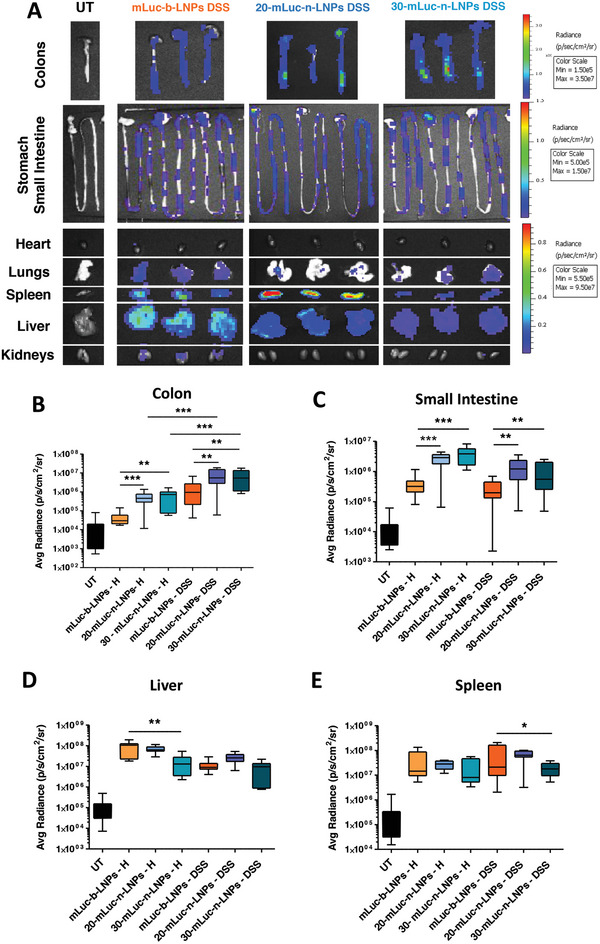
Biodistribution study in colitic mice. A) Representative pictures of the IVIS analysis performed on DSS mice 6 h post‐injection of mLuc‐b‐LNPs and mLuc‐n‐LNPs, and their relative radiance quantification for the colons (B), stomach and small intestines (C), Livers (D) and Spleens (E) (12 mice/group, data are presented as Box and Whiskers from minimum to maximum. Statistical analysis was performed using one‐way ANOVA; ^*^ *p* < 0.05, ^**^ *p* < 0.01, ^***^ *p*,0.001).

To test the in vivo delivery of n‐LNPs compared to b‐LNPs, healthy (H) or DSS colitis‐bearing mice (DSS) induced as previously shown^[^ ^39^, ^40^, ^41^, ^42^ ^]^ were established showing consistent body weight loss and colon shortening (Figures S8A,B, Supporting Information). Next, mice were injected i.v. with mLuc‐b‐LNPs, mLuc‐20‐n‐LNPs, or mLuc‐30‐n‐LNPs at a dose of 10 µg of mRNA per mouse. After 6 h, mice were sacrificed, and their organs were harvested. Luminescence induced by mLuc transfection was measured via IVIS. As presented in **Figures** **2**A and S9A (Supporting Information), and their relative quantification (Figures 2B–E; Figures S9 B–D, Supporting Information), mLuc‐20‐n‐LNPs and mLuc‐30‐n‐LNPs showed higher colon accumulation in healthy animals compared to b‐LNPs. Importantly, the new formulations elicited significantly higher Luc signal in the inflamed colons than mLuc‐b‐LNPs in both healthy (Figure 2B, 5.7 ± 1.2·10^5^ units for mLuc‐20‐n‐LNPs, and 6.1 ± 1.4·10^5^ units for mLuc‐30‐n‐LNPs, vs 0.48 ± 0.12·10^5^ units for b‐LNPs) and in DSS colitis‐bearing mice (7.89 ± 2.1·10^6^ for mLuc‐20‐n‐LNPs, 7.1 ± 1.8·10^6^ units for mLuc‐30‐n‐LNPs, and 1.8 ± 0.6·10^6^ units, respectively). In the small intestine (Figure 2C), mLuc‐20‐n‐LNPs (2.7 ± 1.3·10^6^ units) and mLuc‐30‐n‐LNPs (3.8 ± 0.6·10^6^ units) still showed higher Luc transfection compared to mLuc‐b‐LNPs in healthy mice (3.7 ± 0.8·10^5^ units), with slightly lower signals in DSS mice for all formulations (1.5 ± 0.4·10^6^ units, and 1.1 ± 0.3·10^6^ units vs 2.7 ± 0.6·10^5^ units respectively). Despite the small intestine not being the primary site of action for DSS colitis, this intestinal segment is still somewhat affected by DSS treatment, showing increased immune cell accumulation and expression of pro‐inflammatory cytokines,^[^ ^43^ ^]^ but no increase in Luc signal was observed in DSS mice compared to healthy animals. Thus, these results seem to point to some level of natural colon tropism of the 30‐n‐LNPs formulation especially in inflammatory conditions.

A significant reduction in mice livers transfection was seen for mLuc‐30‐n‐LNPs compared to mLuc‐b‐LNPs, in healthy mice, while there was a non‐significant decrease in DSS mice (Figure 2D). Conversely, the signal in the inflamed livers was slightly lower compared to their healthy counterparts. This could be caused by the general inflammatory state present in DSS‐treated mice that could reduce mRNA expression levels. Indeed, as summarized in Figure S10 (Supporting Information), Luc levels generally decreased in DSS mice when compared to healthy animals, with the decrease observed across different LNPs formulations, but proportional to the amount of DSS concentration, highlighting the negative effect of increased inflammation on Luc transfection.

Reduction in spleen uptake was observed for mLuc‐30‐n‐LNPs compared to mLuc‐b‐LNPs (Figure 2E), although the difference was significant only in the DSS‐inflamed mice.

The colon specificity of mLuc‐30‐n‐LNPs is underlined by a higher Luc signal colon/liver ratio of 0.836 ± 0.109 compared to 0.389 ± 0.101 for mLuc‐20‐n‐LNPs and 0.179 ± 0.064 for mLuc‐b‐LNPs (Figure S11A, Supporting Information). A similar trend was also noticeable for the ratio between colon and spleen in colitis‐bearing mice, although the difference was not significant (Figure S11B, Supporting Information, *p* = 0.086). A similar trend was also observed when presenting the data as fractions of radiance for each formulation (Figure S12, Supporting Information).

Finally, no clear trend or significant differences in Luc signal were visible in the other organs tested, mostly because of the overall low transfection efficiency in these tissues (Figures S9B–D, Supporting Information).

In summary, 30‐n‐LNPs demonstrated a natural tropism toward the inflamed colon while displaying improved evasion of well‐known mononuclear phagocytic system‐associated organs such as the liver and the spleen. Thus, we selected 30‐n‐LNPs as the lead formulation for further testing.

To confirm the results observed with Lipid 15, we also formulated LNPs using the commercially available lipid SM‐102 (SM‐102‐b‐LNPs and SM‐102‐30‐n‐LNPs). As summarized in Figure S13A (Supporting Information), these LNPs displayed a similar size and PDI to the particles prepared with Lipid 15 and also showed good mRNA encapsulation and retention (Figures S13B,C, Supporting Information). When tested for their organ biodistribution in vivo in DSS‐colitis‐bearing mice (Figures S13D,E, Supporting Information), SM‐102‐ LNPs showed a very similar pattern in the colon with n‐LNPs accumulating more, with a heightened specificity for DSS mice (Figures S13F,G, Supporting Information). A very similar pattern to the previous result was also observed in the small intestine in which n‐LNPs accumulated more, with no difference between healthy and DSS colitis‐bearing mice. However, SM‐102‐30‐n‐LNPs induced higher levels of Luc expression in the liver and the spleen in comparison to the LNPs containing Lipid 15. The other organs displayed a much lower signal, with 30‐n‐LNPs generally showing higher levels of transfection.

Taken together, these results show how 30‐n‐LNPs can reliably target the colon and small intestine better than b‐LNPs, albeit the distribution in other organs can be influenced by the ionizable lipid itself, suggesting a sophisticated interplay between LNPs formulation and composition. Due to the superior performance of Lipid 15 in terms of liver and spleen evasion, we focused on this formulation for further investigation.

### 2.4 In Vivo Cellular Uptake of LNPs

Next, we examined, which cells uptake the LNPs within specific organs. We injected intravenously into DSS colitis‐bearing mice a dose of LNPs equivalent to 20 µg of Cre mRNA (mCre) and sacrificed the mice 48 h post‐injection. Their colons, mesenteric lymph nodes, livers, and spleens were harvested and were processed as previously described^[^ ^43^ ^]^ main sub‐populations transfected by mCre‐b‐LNPs or mCre‐30‐n‐LNPs were assessed by flow cytometry.

**Figure 3:**
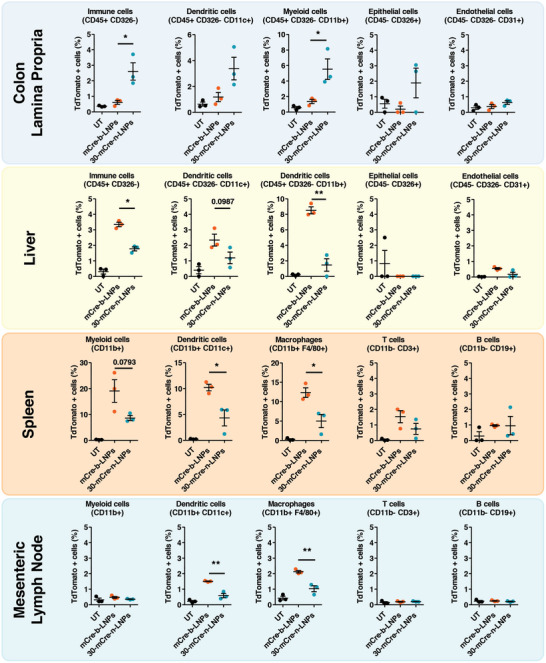
Flow cytometry profiles of the tdTomato^+^ cells in the main cell populations were analyzed for the mice colon lamina propria, liver, spleen, and mesenteric lymph nodes (three mice were included in each experimental group. All data are presented as average ± SEM; Statistical analysis was performed using one‐way ANOVA; ^*^ *p* < 0.05, ^**^ *p* < 0.01).

As displayed in **Figure** **3**, the LNPs in the colon lamina propria transfected mostly immune cells (CD45^+^ CD326^−^). The most notable transfection was visible in the CD45^+^ CD326^−^ CD11b^+^cells populations, with ≈5% of these cells being positive after mCre‐30‐n‐LNPs treatment, compared to the 1.5% of mCre‐b‐LNPs. Dendritic cells (CD45^+^ CD326^−^ CD11c^+^) showed a similar trend, albeit the increased signal elicited by mCre‐n‐LNPs was not statistically significant. This suggests that the majority of the tdTomato signal is derived from other myeloid populations such as monocytes and macrophages, but also possibly the B‐2 cell population present in the gut.^[^ ^44^, ^45^ ^]^ Conversely, no clear trend was evidenced in epithelial (CD45^−^ CD326^+^) and endothelial (CD45^−^ CD326^−^ CD31^+^) cells.

Taken together, these results seem to consolidate our previous observations in the mLuc biodistribution, with mCre‐30‐n‐LNPs showing higher transfection than the benchmark. Moreover, the expression mediated mostly by myeloid cells highlights how these leukocytes, which accumulate in the colon during colitis, are pivotal to the observed 30‐n‐LNPs specificity, confirming the contribution of inflammation to the transfection of these new LNPs.

Moving the focus to the liver, an opposite trend is observed, with the only notable uptake being once again observed in local immune and specifically myeloid and dendritic cells. However, in this instance, mCre‐b‐LNPs appear to transfect these cells significantly more than mCre‐n‐LNPs, with over 3% of immune cells being positive for b‐LNPs compared to less than 2% for 30‐n‐LNPs, and 2.6% of dendritic and 8% of myeloid cells being positive for the benchmark particles, compared to around less than 2% for the new formulation in both populations. No relevant uptake was observed in epithelial or endothelial cells. This also confirms our previous observation, with mCre‐30‐n‐LNPs avoiding liver uptake and clearance from circulation.

Similarly, the analysis of the spleen immune population revealed how mCre‐30‐n‐LNPs transfect significantly fewer myeloid cells in general (CD11b^+^) and dendritic cells (CD11b^+^ CD11c^+^) and macrophages (CD11b^+^ F4/80^+^) in particular. This confirms the 30‐n‐LNPs’ ability to escape uptake from tissue‐resident phagocytic cells. Interestingly, another similar trend is observed in the non‐myeloid cells (CD11b^−^), which was less transfected by mCre‐30‐n‐LNPs compared to mCre‐b‐LNPs, and in particular B cells (CD11b^−^ CD19^+^). Therefore, it appears that mCre‐30‐n‐LNPs are not only able to escape undesired phagocytic uptake but also uptake from lymphoid cells.

Finally, the mesenteric lymph nodes displayed a similar pattern to the spleen in the myeloid population, in which fewer cells were transfected by mCre‐30‐n‐LNPs compared to mCre‐b‐LNPs.

These results not only confirm our previous data but also elucidate how the mechanism of 30‐n‐LNPs selective transfection of the inflamed colon is driven by both the accumulation of infiltrated myeloid cells in the colon, while the same time avoiding uptake by mononuclear phagocytic cells in the liver, spleen, and even in the mesenteric lymph nodes.

To suggest a possible mechanism behind the 30‐n‐LNPs targeting colon inflammation, we investigated their interactions with blood leukocytes. Thus, healthy or DSS mice were injected i.v. with Cy5‐labelled RNA loaded b‐LNPs (Cy5‐b‐LNPs) or 30‐n‐LNPs (Cy5‐30‐n‐LNPs). Cy5‐b‐LNPs and Cy5‐30‐n‐LNPs had analogous size, PDI, and RNA encapsulation to their Luc‐loaded analogs (Figures S14A,B, Supporting Information). Three hours after the injection, mice were bled and their blood leukocytes were analyzed by flow cytometry. DSS mice blood presented a higher amount of monocitic‐myeloid derived suppressor cells (MDSCs, CD11b^+^ Ly6C^+^ Ly6G^−^) and polymorph nuclear‐MDSCs (CD11b^+^ Ly6C^+^ Ly6G^+^), which are normally elevated in colitis (Figure S14C, Supporting Information).^[^ ^46^, ^47^ ^]^ Notably, Cy5‐30‐n‐LNPs interacted more than Cy5‐b‐LNPs with T cells (CD11b^−^ CD3ε^+^) and monocytic‐MDCSs both in healthy and colitic animals and more with polymorph nuclear‐MDSCs only in healthy mice (Figure S14D, Supporting Information).

Compiling this evidence with our previous results, we can hypothesize that 30‐n‐LNPs interact strongly with these myeloid cells in the bloodstream, and be transported by them to the colon, especially during colonic inflammation which mobilizes MDCSs in the blood.

### 2.5 n‐LNPs Entrapping IL‐10 mRNA Show Improved Therapeutic Outcomes in Inflamed Mice

To test the therapeutic efficacy of our LNPs formulation, murine IL‐10 mRNA was encapsulated in b‐LNPs or 30‐n‐LNPs. These LNPs were characterized using DLS and RiboGreen, showing analogous features to their respective mLuc formulations (Figures S15A,B, Supporting Information). DSS colitis‐bearing mice were injected retro‐orbitally with LNPs dose equivalent to 20 µg of either mIL‐10‐b‐LNPs or mIL‐10‐30‐n‐LNPs on days 3, 5, and 7 from the start of DSS exposure. On day 8, mice were sacrificed for analysis.

**Figure 4:**
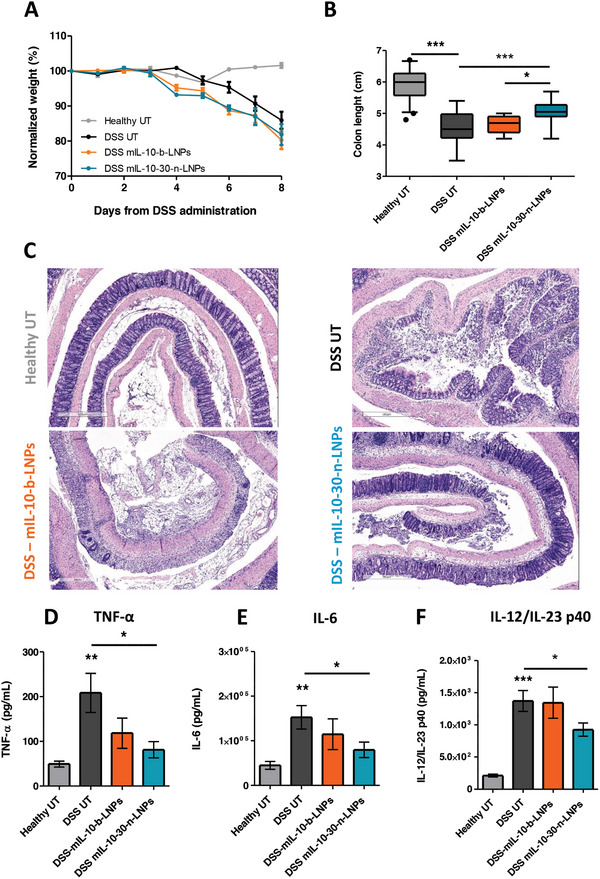
Improved therapeutic outcome in colitic mice with IL‐10 mRNA delivered with n‐LNPs. Assessment of the mouse body weight (A) and colon length (B) during and after treatment with LNPs, respectively (*n* = 15 mice/group). C) representative hematoxylin/eosin histological sections of rolled mice colons. ELISA measurement of the colon levels of TNF‐α (D), IL‐6 (E), and IL‐12/IL‐23 p40 (F). 10 mice were included in each group; 5 mice were included for mIL‐10‐b‐LNPs. (All data are expressed as average ± SEM; Statistical analysis was performed using a one‐way ANOVA test ^*^ *p* < 0.05, ^**^ *p* < 0.01, ^***^ *p* < 0.001.)

As Presented in **Figure** **4**A, the mouse body weight loss was comparable across all the DSS‐exposed experimental groups, with no apparent benefit from the administration of mIL‐10‐loaded‐LNPs. However, this apparent lack of benefit could be attributed to the animals avoiding drinking water containing DSS, resulting in weight loss caused by dehydration. This has already been observed in similar studies.^[^ ^40^ ^]^

Mice colon length after treatment was also assessed (Figure 4B), revealing how comparing untreated DSS mice (DSS UT, 4.56 ± 0.12 cm) with mice that received the mIL‐10‐30‐n‐LNPs displayed a significant benefit by partially preserving colon length (5.06 ± 0.09 cm), with no protective effect exerted by the control mIL‐10‐b‐LNPs (4.66 ± 0.29 cm).

To further examine the efficacy results, colon tissues were harvested and sectioned for histological analysis. As evidenced by the representative pictures in Figure 4C, colon sections from healthy mice demonstrated well‐structured tissues with clear crypts and lamina propria. However, DSS untreated (UT), as well as colitic animals treated with mIL‐10‐b‐LNPs revealed extensive epithelial damage, with compromised barrier integrity, loss of crypt structure, and less clear separation from the epithelial layer and lamina propria, which showed high levels of leukocytes accumulation. On the other hand, mIL‐10‐30‐n‐LNPs showed the ability to partially protect the colon integrity, preserving most of the epithelial integrity and crypt structure and decreasing immune cell infiltration into the lamina propria.

After measuring the colons, the expression of key cytokines was also assessed in the tissue. Tumor necrosis factor alpha (TNF‐α) is considered a major driver of inflammation in IBDs, to the point that the first recombinant antibody used to treat colitis, infliximab, was designed to block and remove this cytokine.^[^ ^48^ ^]^ Notably, as presented in Figure 4D, TNF‐α was significantly increased in DSS UT mice (208.3 ± 138.5 pg mL^−1^), and only the treatment with mIL‐10‐30‐n‐LNPs resulted in a significant decrease in its concentration (81.2 ± 54.39 pg mL^−1^).

Interleukin 6 (IL‐6) is another pro‐inflammatory cytokine involved in a variety of immune responses. In IBDs, IL‐6 contributes to the loss of the intestinal barrier function by increased colon permeability, enabling bacterial penetration into the tissue, and has an anti‐apoptotic effect on T cells, contributing to the sustained inflammatory environment.^[^ ^49^ ^]^ The viability of IL‐6 as a therapeutic target is still being investigated with promising results against Crohn's disease by using recombinant soluble IL‐6R as decoys. Indeed, treatment with mIL‐10‐30‐n‐LNPs significantly decreased IL‐6 levels compared to DSS UT (79700 ± 54524 pg mL^−1^ and 152517 ± 82500 pg mL^−1^, respectively, Figure 4E).

Similarly, the subunit p40 shared by the inflammatory cytokines IL‐12 and IL‐23 (IL‐12/IL‐23 p40) has also been identified as an important endpoint for the assessment of colitis,^[^ ^50^ ^]^ and has been validated as a therapeutic target for the monoclonal antibody Ustekinumab. Remarkably, the elevated levels of p40 measured in DSS UT animals (1372 ± 514 pg mL^−1^) were significantly reduced by the administration of mIL‐10‐30‐n‐LNPs (927 ± 237.9 pg mL^−1^ Figure 4F).

Taken together, these results show that on the macroscopic, histological, and molecular levels, mIL‐10‐30‐n‐LNPs can exert a protective effect on mice colons against DSS‐induced tissue damage and inflammation.

### 2.6 In Vivo Safety Profile of LNPs

**Figure 5:**
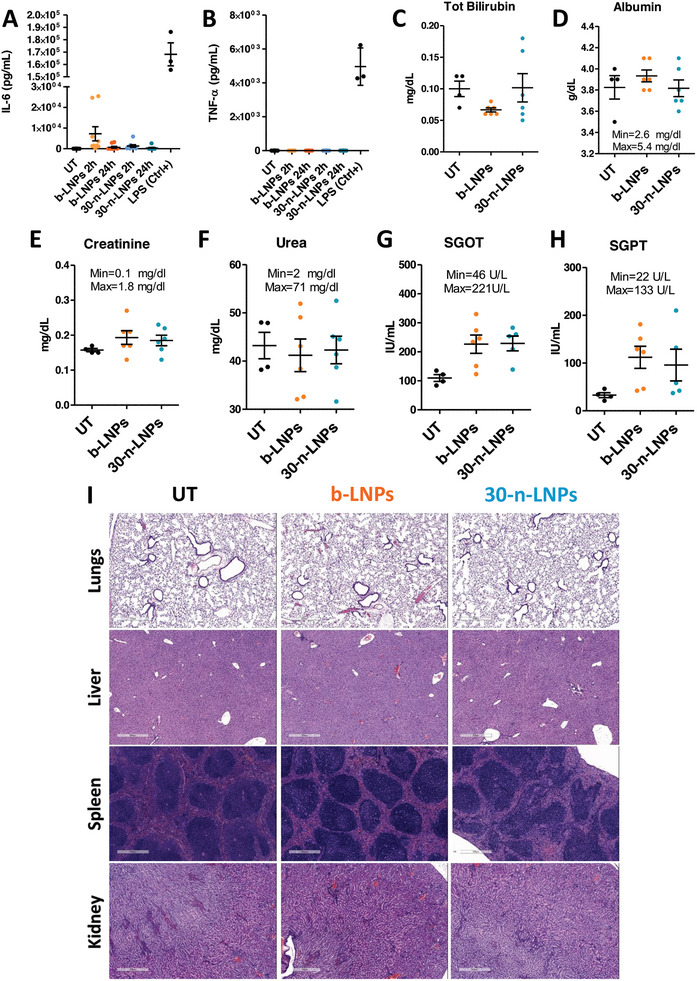
Toxicity profile of LNPs. Assessment of the plasma levels of IL‐6 (A) and TNF‐α (B) 2 and 24 h after injection of mLuc‐b‐LNPs, mLuc‐n‐nLNPs, or LPS (*n* = 6 mice/group). Measurement of the blood levels of total bilirubin (C), albumin (D), creatinine (E), Urea (F), SGOT (G), and SGPT (H) (*n* = 5 or 6 mice/group). Min and Max values represent the normal range for each marker, as adapted from the literature.^[^ ^51^ ^]^ (I) representative images of Hematoxylin/Eosin‐stained mouse Lungs, Livers, Spleens, and Kidneys after treatment with LNPs. Scale bars equivalent to 500 µm. Data are presented as average ± SEM.

To assess the in vivo safety of b‐LNPs and 30‐n‐LNPs, we injected mLuc‐LNPs intravenously at a dose of 20 µg of mRNA per animal. Mice were then bled after 2 or 24 h from administration. After isolating the animals’ plasma, levels of TNF‐α and IL‐6 were measured by ELISA. As a positive control for acute inflammation, mice were administered i.v. with 10 µg of LPS dissolved in sterile 1X PBS. As displayed in **Figure** **5**A, while LPS elicited very high cytokine levels, both formulations led to far lower levels of cytokines, with IL‐6 showing less than 10‐fold protein in comparison, and no TNF‐α whatsoever (Figure 5B) 2 h post i.v. administration. IL‐6 levels reduced to normal levels 24 h post‐injection. Taken together, these results show that the 30‐n‐LNPs formulation did not lead to an immune response and can be considered safe in this context.

To get a better picture of LNPs possible adverse effects upon intravenous administration, the plasma collected 24 h after administration was also tested for a wide variety of biochemical markers. According to the results summarized in Figures 5C–F, the two formulations did not cause significant changes in the blood levels of total bilirubin and albumin, creatinine, and urea.

Liver enzyme levels such as serum glutamic oxaloacetic transaminase (SGOT, Figure 5G), and glutamic pyruvic transaminase (SGPT, Figure 5H) were slightly altered compared to untreated mice but are still within the acceptable physiological levels reported for C57BL/6 mice in the literature.^[^ ^49^ ^]^ Similarly, all other markers tested were found to be unaltered by LNPs injection (Figure S16, Supporting Information).

Finally, mice livers, spleens, lungs, and kidneys were harvested after 24 h from injection for histological assessment. As displayed in Figure 5I, no signs of immune cell infiltration or tissue damage were visible across any of these organs compared to the untreated mice.

These results suggest that both tested LNPs formulations are generally well tolerated and that this tolerance is not dependent on LNPs composition.

## 3 Conclusions

In the present work, we assessed the effect of changes in the helper lipid amounts in LNPs formulations on their in vivo behavior upon systemic administration. We described how n‐LNPs, characterized by a lower amount of ionizable lipid and cholesterol but a higher percentage of helper lipid (20 or 30% DSPC) compared to b‐LNPs resulted in LNPs with a slightly smaller size and ζ potential. These particles still resulted in homogeneous size distribution, had optimal mRNA encapsulation, and were able to induce mLuc and IL‐10 expression in vitro across multiple cell lines.

When assessing the transfection efficiency of these particles in vivo, we demonstrated how 30‐n‐LNPs induced a highly selective Luc expression in the inflamed colons of DSS colitic mice, while at the same time de‐targeting the liver and spleen. Further investigation of the cells involved in this tropism demonstrated how the main source of mRNA expression were myeloid cells, and most likely monocytes and macrophages that accumulated into the colon lamina propria and epithelium during colitis, or B‐2 cells that are known to be present in this tissue and that are believed to have the potential to ameliorate colitis.^[^ ^44^, ^45^ ^]^ Indeed, it has been shown that the colon is characterized by peculiar immune trafficking, both in the healthy steady state and in inflammation. In particular, the intestine is considered one of the few tissues in which the presence of local phagocytic cells is strongly dependent on the constant migration of circulatory monocytes to the lamina propria, which then differentiate into dendritic cells and macrophages. This process is accelerated during colitis.^[^ ^52^, ^53^, ^54^ ^]^ Furthermore, our evidence shows how 30‐n‐LNPs interact more with MDCSs compared to b‐LNPs in the blood of both healthy and colitic mice. Thus, the observed tropism of 30‐n‐LNPs may be mediated by these particle's ability to target circulating monocytes and “piggyback” them to the inflamed colon, albeit further studies would be required to both confirm this hypothesis and understand the molecular mechanism behind this preferential interaction of 30‐n‐LNPs compared to b‐LNPs. Furthermore, 30‐n‐LNPs were well tolerated after a single i.v. bolus administration in vivo.

When testing the viability of n‐LNPs as therapeutic candidates, we loaded this formulation with mRNA encoding for the anti‐inflammatory cytokine IL‐10, which has previously shown the potential to re‐establish the immune homeostasis in the inflamed colon by reducing the accumulation of M1‐monocytes and pro‐inflammatory cells such as Th_1_ and Th_17_ lymphocytes and promoting the differentiation of immunosuppressive populations such as T_reg_ lymphocytes.^[^ ^55^ ^]^ Indeed, we demonstrated how treatment with mIL‐10‐30‐n‐LNPs during the induction of colitis had a protective effect on the mice intestines and preserved the colon epithelium barrier and crypt structure, with reduced immune cell infiltration. This was similarly related to a decrease in the levels of pivotal inflammatory cytokines including TNF‐α, IL‐6, and the p40 subunit shared by IL‐12 and IL‐23.

Ultimately, these results highlight how the fine‐tuning of the LNP's composition can radically influence their biological behavior. Compared to using different lipids, modulating particle size, and surface charge, or using active targeting moieties, simply changing the LNP's composition appears a simple yet elegant tool to modify the LNPs’ biodistribution that does not require the cumbersome steps of chemical synthesis and purification, nor the labor‐intensive and costly techniques of chemical conjugation or recombinant proteins expression. Thus, this study expands our current toolbox of LNP's formulations and underlines how it can be expanded to organs that are not often the target of mRNA therapeutics, such as the gastrointestinal tract.

These types of formulations open new frontiers, exploring the ideal LNPs composition to target different tissues and different pathologies and their underlying targeting mechanism.

## 4 Experimental Section

### Chemicals and Cell Culture

1,2‐distearoyl‐sn‐glycero‐3‐phosphocholine (DSPC,Cat# 850365P‐1g), cholesterol (Cat# 700000P‐5g), and 1,2‐dimyristoyl‐rac‐glycero‐3‐methoxypolyethylene glycol‐2000 (PEG‐DMG 2000, Cat# 880151P‐5g) and SM‐102 were purchased from Avanti Polar Lipids. Ionizable Lipid (Lipid 15) was produced and purified in‐house following an established synthetic route as previously described. Chemical structures for the ionizable lipids are presented in Figure S1 (Supporting Information). Ethanol absolute (Cat# 000525052100) and 2‐propanol (Cat# 001626052100) were provided by Bio‐Lab. DPBS 1X was purchased from Gibco (Cat# 14190‐169), PBS 10X from Hylabs (Cat# BP507/500D), DEPC‐treated water from BioPrep (Cat# DPH20‐500ML), and 0.5 m Citrate buffer solution from Thermo Scientific (pH = 4.5, Cat# J60024.AK). Modified Luciferase mRNA was provided by BioNtec, and EGFP mRNA was purchased from TriLink (Cat#L‐7601‐1000).

Fetal Bovine Serum was purchased from Biowest (FBS, heat‐inactivated, EU origin, Cat# S140H‐500). L‐Glutamine 200mm (100X, Cat# 25030‐024), 0.25% Trypsin‐EDTA (1X, Cat# 25200‐114), Penicillin‐Streptomycin solution (10,000 U mL^−1^ of Penicillin + 10000 µg mL^−1^ of Streptomycin, Cat# 15140‐122), DMEM (1X, Cat# 41965‐039), and RPMI Medium 1640 (1X, Cat# 21875‐034) were provided from Gibco.

HeLa cells (Cat# CCL‐2), RAW264.7 cells (Cat# TIB‐71), HepG2 cells (Cat# HB‐8065), Caco‐2 (Cat# HTB‐37) and HCT‐116 cells (Cat# CCL‐247) were provided from ATCC. Cell cultures were maintained in an incubator at 37 °C in a controlled atmosphere (5% CO2, 95% humidity) using the culture media required by the producer using T25 and T75 flasks.

### mRNAs

Luciferase mRNA was kindly provided by BioNtech. Cre mRNA was purchased from GeneScript.

Custom‐modified mouse IL‐10 mRNA was purchased from TriLink with the following ORF:

5′_ATGCCTGGCTCAGCACTGCTATGCTGCCTGCTCTTACTGACTGGCATGAGGATCAGCAGGGGCCAGTACAGCCGGGAAGACAATAACTGCACCCACTTCCCAGTCGGCCAGAGCCACATGCTCCTAGAGCTGCGGACTGCCTTCAGCCAGGTGAAGACTTTCTTTCAAACAAAGGACCAGCTGGACAACATACTGCTAACCGACTCCTTAATGCAGGACTTTAAGGGTTACTTGGGTTGCCAAGCCTTATCGGAAATGATCCAGTTTTACCTGGTAGAAGTGATGCCCCAGGCAGAGAAGCATGGCCCAGAAATCAAGGAGCATTTGAATTCCCTGGGTGAGAAGCTGAAGACCCTCAGGATGCGGCTGAGGCGCTGTCATCGATTTCTCCCCTGTGAAAATAAGAGCAAGGCAGTGGAGCAGGTGAAGAGTGATTTTAATAAGCTCCAAGACCAAGGTGTCTACAAGGCCATGAATGAATTTGACATCTTCATCAACTGCATAGAAGCATACATGATGATCAAAATGAAAAGCTAA‐3′

The UTRs and Poly‐A tail were optimized by the producer.

### LNPs Formulation

LNPs were formulated using a NanoAssemblr Benchtop (Cat# NIT0055) equipped with a heating block (Cat# NIT0026) and its relative cartridges (Cat# NIS0009), purchased from Precision NanoSystems Inc. (Vancouver, Canada).

To prepare the LNPs organic phase, lipids were mixed following two possible combinations of lipids molar ratios: 50% amino ionizable, 38.5% Cholesterol, 1.5% PEG‐DMG, 10% DSPC for b‐LNPs; 44% amino ionizable lipid, 34.3% Cholesterol, 20% DSPC, 1.75% PEG‐DMG for 20‐n‐LNPs; or 38% amino ionizable, 30% Cholesterol, 30% DSPC, 2% PEG‐DMG for 30‐n‐LNPs. Lipids were combined to a final concentration of 6mm. The lipid components were dissolved in absolute ethanol and heated to 55 °C. The aqueous phase was composed of 25 mm citrate buffer (pH = 4.5, ThermoScientific, Cat# J60024.AK) containing the RNA of interest. pH was checked using a pH indicator (Cat# 1.09535, Supelco). The N/P ratio of Lipid 15 to mRNA was 12. The lipid phase and aqueous phase were loaded in the NanoAssembler in 1 mL and 3 mL syringes (BD), respectively. The particles were assembled at 55 °C, using a flow rate ratio (FRR) of 3:1 (aqueous: ethanol), a total flow rate (TFR) of 12 mL min^−1^, and a pre‐and post‐waste of 50 µL. Particles were subsequently dialyzed using MAXI GeBaFlex‐tubes, 14kDa MWCO (Gene Bio‐Application LTD, Cat# D050‐100), against 0.5X PBS for 3 h, followed by overnight dialysis against 1X PBS (Hylabs, Cat# BP507/500D).

### LNPs Physical Characterization

LNPs size was assessed by dynamic light scattering (DLS) analysis, using a Zetasizer device (Malvern). LNPs were prepared for size measurement by diluting 10 µL of LNPs in 990 µL of 1X DPBS and measured using a 10 × 4 × 45 mm Polystyrene cuvette (Starstedt, Cat#67.742). To estimate the Zeta potential, 10 µL of LNPs were diluted in 990 µL double distilled water (DDW) and loaded in a disposable folded capillary cuvette (Mavlern, Cat# DTS‐1070). Every measurement was performed in triplicate selecting an equilibration time of 1 min and selecting ad hoc diffraction indexes for the used dispersants.

LNPs size and concentration (LNPs/mL) were estimated also by nanoparticles‐tracking analysis (NTA) using a NanoSight NS300 system (Malvern) equipped with an injection pump. LNPs were prepared by diluting them 1:1000 in MilliQ water and loaded in a 1 mL syringe (Pic). Each formulation was measured using a dynamic setting with an injection speed of 100. Five sequential measurements of 60 s each were performed, keeping the camera level at 12. For NTA analysis, the detection threshold was kept at 5 for all measurements.

### TEM Analysis of LNPs

A drop of LNPs aqueous dispersion was dripped on a carbon‐coated copper grid, left to dry, and imaged using a JEOL 1200 EX transmission electron microscope. To assess the LNP's average diameter, pictures taken from TEM were analyzed manually using the FIJI software (Version 1.54i). 250 single nanoparticles were included in each group for quantification.

### Assessment of RNA Encapsulation

mRNA encapsulation in LNPs was assessed using the fluorimetric Quant‐it RiboGreen RNA Assay Kit (Thermo Fisher, Cat# R11490) following the manufacturer's instructions. Samples were diluted in 1X TE buffer, prepared in triplicate, and read on 96 wells plates (Costar, no lid, Black, Flat Bottom, polystyrene, Cat# 3915) and read using a Synergy HT plate reader (BioTek). To induce LNPs disassembly, LNPs were diluted 1:200 in TE buffer 1X mixed with 0.5% (v/v) Triton X‐100 (Merck, Cat# T8787). To estimate the percentage of encapsulated RNA, the Equation (1) was used:

(1) $$E\;\left(\%\right)=\frac{\left(FluoLNPsTr-BlankTr\right)-\;\left(Fluo\;LNPs-Blank\right)}{\left(FluoLNPsTr-BlankTr\right)}*100$$

where E is the encapsulation percentage of mRNA; FluoLNPs and FluoLNPsTr are the fluorescence signals without and with Triton, respectively; Blank and BlankTr are the fluorescence signals of blanks without and with Triton, respectively.

### Agarose Gel Electrophoresis

To confirm the LNPs mRNA encapsulation and retention, volumes of LNPs suspension containing 0.5 µg of mRNA were loaded in a 1% agarose gel containing Midori Green advance DNA stain (Line A, Cat # MG04). To release RNA from LNPs the suspension was mixed with Triton X‐100 to a final detergent concentration of 0.5% (v/v). Every sample was mixed in a 1:1 (v/v) ratio of RNA Loading Dye (2X, Cat# B0363S, New England Biolabs) before being loaded in the gel. The gel was run in the presence of ssRNA Ladder (Cat# N0362S, New England Biolabs) at 100 V for 40 min before being imaged using a Syngene PXi image analysis system.

### Biocompatibility Assay (XTT)

To assess LNPs in vitro biocompatibility, HeLa, RAW264.7, HepG2, or Caco‐2 cells were seeded on 96 wells plates at a density for 5000 cells per well and left to adhere overnight. The day after, cells were treated with decreasing amounts of LNPs (5, 2.5, 1.25, 0.6, 0.3, 0.15, 0.075, 0.038, µg mL^−1^ of RNA). After 72 h from incubation, the cell medium was supplemented with 50 µL of XTT reagent per well (XTT Cell Proliferation Assay Kit, Roche, Cat# 10010200 and left to develop for 2 h, before reading the absorbance at 450 nm using a Syngene PXi image analysis system. The absorbance at 660 nm was used as a reference. Cell viability was normalized by the signal of untreated cells and every measurement was performed in triplicate.

### In Vitro Luciferase mRNA Transfection

HeLa, RAW264.7, HepG2, or Caco‐2 cells were seeded on 96 wells plates at a density of 10000 cells per well and left to adhere overnight. The next day, RAW264.7 cells were stimulated to induce inflammation by exposing them to 100ng mL^−1^ of mouse interferon‐gamma (IFN‐γ, Peprotech, Cat# 315‐05) and lipopolysaccharide (LPS, Sigma–Aldrich). The next day, Luc‐LNPs were added at a concentration of 0.25 µg mL^−1^ of Luciferase mRNA. After 18 h from treatment culture medium was removed and replaced with 50 µL of Luciferase Cell Culture Lysis Reagent (Cat# E1531, Promega) diluted 1:5 in DDW in every well and incubated at RT on an orbital shaker for 10 min. 30 µL of each well was then moved to white plates (Costar, White flat bottom, non‐treated no lid, Cat# 3912). The Luciferase signal was read using a Luciferase Assay System (Cat# E1500, Promega) and a GloMax plate reader equipped with dual injectors. Every well was injected with 50 µL of reagent and incubated for 10 s before reading. Every measurement was performed in quadruplicate.

### Polarization of RAW264.7 Cells

To assess RAW264.7 cell activation, CD64 was selected as a marker for M1 polarization. To this end, RAW264.7 cells were seeded at a density of 3 million cells per well in a six well plate. The next day, cells were stimulated as described above for 24 h. To detect CD64 via SDS‐PAGE/Western blot, cells were lysed using RIPA buffer 1X (Merck Cat#20‐188). Total protein content was quantified using Micro BCA Protein Assay Kit (Thermo Fisher Cat# 23235). Samples were then mixed with Laemmli Sample buffer 1X, 10 µg of total protein per sample were loaded on a 4–20% Mini‐PROTEAN TGX Precast Protein Gel (Biorad, Cat#4561096), and run for 2 h at 120 V. Proteins were transferred to a nitrocellulose membrane overnight at 80 V. Protein transfer was confirmed using a Ponceau S solution (Sigma–Aldrich, Cat# P7170). Membranes were blocked at room temperature for 1h using 1% Difco Skim Milk (BD Life Sciences) in TBST. Membranes were then incubated with an anti‐mouse CD64 rat antibody (Clone AT152‐, Biorad, Cat# MCA5997) at a concentration of 2 µg mL^−1^ for 1 h. After washing with TBST, a secondary, HRP‐conjugated, polyclonal rabbit anti‐rat antibody (OriGene Technologies, Cat# TA130033) was added for 1 h. After further washes, the membrane was analyzed using a Syngene PXi image analysis system.

To detect CD64 using fluorescence microscopy, after seeding and stimulating the cells, they were labeled using either an anti‐mouse PE‐conjugated CD64 antibody (Biolegend, Cat# 161003) or a PE‐conjugated isotype control (Biolegend, Cat# 400607). Cell membranes were labeled using an AlexaFluor 488‐conjugated anti‐mouse CD44 antibody (Biolegend, Cat# 103016) and nuclei using Hoechst solution (Sigma Aldrich, Cat# B2261). CD64 levels were estimated using the FIJI software.

For flow cytometric analysis, cells were stained as described above, using DAPI instead of Hoechst as a marker for cell death, detached, and analyzed using a Cytoflex Flow cytometer (Beckman Coulter).

### In Vitro Assessment of IL‐10 Expression

HeLa, RAW264.7, HepG2, or Caco‐2 cells were seeded at a density of 5000 cells per well in a 96 wells plate. The next day, the RAW 264.7 cells were stimulated with IFN‐γ and LPS as discussed above. After 24 h, cells were treated with 0.5 µg mL^−1^ of IL‐10 mRNA‐loaded LNPs. Cell medium was recovered after 72 h from treatment and stored at −80 °C. Levels of IL‐10 were measured using an ad hoc ELISA kit (mouse IL‐10 DuoSet ELISA, Cat# DY417, R&D systems).

### Assessment of LNPs Stability Under Storage

After their preparation, LNPs were stored in 2 mL clear glass vials (Merck, Cat#27265) at 4 °C. The day after and every 7 days until 28 from formulation, a small aliquot of LNPs was withdrawn and characterized using DLS, Ribogreen, and in vitro Luciferase assay after 24 h as discussed above. As a baseline for transfection in the luciferase assay system, cells were treated with luciferase‐encoding mRNA loaded onto Lipofectamine MessengerMax (ThermoScientific, Cat# LMRNA001), adding 0.1 µg of mRNA per well.

### Establishment of Dextran Sodium Sulfate Colitis Mouse Models

All animal studies were performed in accordance with the protocols approved by the ethics committee (Protocol # TAU‐LS‐IL‐2201‐108‐3). Female, 8 weeks old C57BL/6 mice were treated for 7 days with 2% (m/v) of Dextran sodium sulfate salt (DSS, Colitis Grade, MW = 36000—50000 Da Cat# MFCD00081551, MP Biomedicals) administered ad libitum in the drinking water. DSS was replaced with a fresh solution on day 3 from the treatment start. Mice's body weight was assessed at the beginning of DSS exposure and then every day until day 7. If a mouse met the humane endpoints previously established (10% body weight loss in 24 h or 20% body weight loss since the beginning of the experiment), it would be immediately excluded from the study and euthanized.

### Assessment of In Vivo LNPs Biocompatibility

LNPs biocompatibility was assessed by administering the LNPs i.v. Retro‐orbitally at a dose of 20 µg of mRNA per mouse. Every treatment group included 3 healthy, 8–10 weeks old, female C57BL/6 mice. After 2 or 24 h from treatment, mice were terminally bled under Ketamine/Xylazine anesthesia and sacrificed, and their lungs, livers, spleens, and kidneys were harvested and fixed in 4% formalin and stored at 4 °C. The mice plasmas were separated from whole blood using BD Microtainer SST Blood collection tubes containing clot activator (BD, Cat# 365968) and centrifuged at 3500rpm for 10 min using a fixed‐angle rotor centrifuge. Plasmas were then analyzed for IL‐6, TNF‐α, and MCP‐9 using ad hoc ELISA kits. Blood chemistry analysis was performed by AML Lab Services, focusing on the quantification of SGOT, SGPT, LDH, GGTP, CPK, alkaline phosphatase, bilirubin, creatinine, urea, total protein, albumin, globulin, triglycerides, cholesterol, sodium, calcium, phosphates, glucose, chloride, and potassium blood levels. Histological analysis was performed by Histospeck by including the tissues in paraffin, sectioning them, and performing hematoxylin/eosin staining. For both blood chemistry and histological assessment, tissue samples were labeled to make the final analysis blinded.

### LNPs Organ Distribution In Vivo

DSS colitis‐bearing mice were injected intravenously retro‐orbitally with Luciferase mRNA‐loaded LNPs at a dose of 10 µg of mRNA per mouse. After 6 h from the injection, mice were injected intraperitoneally with 200 µl of IVISBrite D‐luciferin Potassium Salt 15 mg mL^−1^ (PerkinElmer Cat#122799) and anesthetized by isoflurane inhalation. After 5 min, mice were sacrificed by cervical dislocation, and their lungs, hearts, livers, spleens, stomach small intestines, colons, and kidneys were harvested. Organs were then imaged using an IVIS Lumina system selecting automatic focus and exposure time to detect the chemiluminescence signal. The average radiance for each organ was measured using the Living Image 4.1 Software using ad hoc ROIs. Radiance is expressed as photons/sec/cm^2^/sr (from now on referred to as radiance units).

### Assessment of LNPs Cellular Transfection in Cre‐tdTomato Mice

DSS colitis‐bearing mice were established as discussed above using B6g.Cg‐Gt(ROSA)26Sortm9 (CAG‐tdTomato)/Hze/j mice (from now on referred to as Cre‐tdTomato mice for brevity). At day 6 from DSS exposure, the mice were treated with b‐LNPs or 30‐n‐LNPs at a dose of 20 µg of Cre mRNA via retro‐orbital vein injection. For this application, LNPs were loaded with mRNA encoding for the Cre recombinase enzyme. After 48 h, the mice were sacrificed and colons and mesenteric lymph nodes (C1), as well as livers and spleens, were harvested.

Colons were isolated from their caeca. Then, colons and mesenteric lymph nodes were stored in an ice‐cold complete medium (RPMI supplemented with 25 mm HEPES, 1 mm sodium pyruvate, 1% Pen/Strep, and 2% Glutamine) for processing. The fat was manually removed from the intestine before opening it longitudinally, removing the fecal matter by wash in ice‐cold PBS and cut in pieces. The tissues were then washed in DTT wash solution (HBSS ‐/‐/‐ supplemented with 25 mm HEPES, 1 mm sodium pyruvate, 1x MEM‐NEAA, and 1mM DTT) and left for 10 min under shaking at room temperature (RT). The DTT wash was then replaced with EDTA wash buffer (HBSS ‐/‐/‐ supplemented with 25 mm HEPES, 1 mm sodium pyruvate 1x MEM‐NEAA, and 5 mM EDTA) and left under shaking for 10 min at RT. This process was repeated twice more. Every wash fraction was saved to remove the epithelial cells (epithelial fraction). Tissues were then digested by incubating them at 37 °C in Digestion buffer (complete medium supplemented with DNAse I 0.1 mg mL^−1^, Dispase 0.1 µ mL^−1^, Collagenase D 1 mg mL^−1^, 0.1 m DTT) for 1 h after manual mincing at 37 °C. After digestion, the tissue was passed through 70 µm strainers, and the suspended cells were then pelleted by centrifugation at 2000 rpm for 7 min. Cells were then resuspended in 40% Percoll solution and 80% Percoll was carefully added underneath the cell suspension. The gradient was then centrifuged at 2200 rpm for 22 min, 22 °C. After removing the samples from the centrifuge, the immune cell layers were carefully recovered and resuspended in complete media.

In parallel, spleens and lymph nodes were ruptured using 70 µm nylon strainers to release the cells and centrifuged at 300 g for 5 min at 4 °C. After removing the supernatant, cells were then resuspended in red blood cells lysis buffer (Sigma–Aldrich) for 2 min before being quenched with 10 mL of complete DMEM, and centrifuged again at 300 g for 5 min at 4 °C. Finally, after decanting the supernatant, cells were resuspended in 0.5 mL of complete DMEM for counting and staining.

Livers were processed using the Miltenyi mouse liver dissociation kit (Cat# 130‐105‐807) following the manufacturer's instructions.

For each mouse, the mesenteric lymph nodes, colon epithelial fraction, colon lamina propria, livers, and spleens cells were counted and a volume equal to 1 million cells was transferred in FACS tubes. Cells were then treated with mouse FcR blocker reagent (Miltenyi) according to the manufacturer's instructions and then stained with different antibody panels as reported in **Tables** **1** and **2**.

**Table 1:** Antibody panel used to stain cell population in mice lamina propria, epithelial fraction, and livers.

Antigen^)^	Fluorophore	Producer	Catalog #	Concentration [mg mL^−1^]	Amount/million cells [µg]
CD45	APC‐Fire 750	Biolegend	147714	0.2	0.125
CD31	AlexaFluor488	Biolegend	102414	0.5	1
CD326	APC	Biolegend	118213	0.2	0.125
CD11b	PE‐Cy7	Biolegend	101215	0.2	0.125
CD11c	PerCP	Biolegend	117325	0.2	0.125

**Table 2:** Antibody panel used to stain cell population in mice mesenteric lymph nodes and spleens.

Antigen^)^	Fluorophore	Producer	Catalog #	Concentration [mg mL^−1^]	Amount/million cells [µg]
CD3e	PerCP	Biolegend	100325	0.2	0.5
CD19	FITC	Biolegend	152404	0.5	0.0625
CD11b	APC‐Cy7	Biolegend	101226	0.2	0.125
CD11c	APC	Biolegend	117309	0.2	0.125
F4/80	PE‐Cy7	Biolegend	123113	0.2	0.125

After 30 min of incubation at 4 °C, antibodies were quenched by adding 2 mL of FACS buffer and centrifuged at 500*g* for 5 min. Supernatants were removed and cells were finally resuspended in 100 µL of FACS buffer supplemented with DAPI 5 µg mL^−1^. Cells were then analyzed using a Cytoflex Flow cytometer using the gating strategy summarized in Figure S17 (Supporting Information).

### Assessment of LNPs’ Interaction with Blood Leukocytes

Healthy or colitis‐bearing mice were injected intravenously with LNPs loaded with a 50:50 weight ratio of Luc mRNA and Cy‐5 labeled, negative control siRNA at a dose of 10 µg of total RNA. 3 h after injection, mice were terminally bled and the blood gathered in BD Microtainer K2EDTA 250–500 µL tubes. 500 µL of blood were treated with 2 mL of Red blood cells were lysed using Red Blood Cell Lysing Buffer Hybri‐Max (Sigma–Aldrich) for 2 min and then quenched with 20 mL of 1XPBS. The cells were then centrifuged at 300*g* for 5 min and resuspended in FACS buffer. The cellular suspension was then treated with a mouse FcR blocking agent (Miltenyi), before being stained with the antibody panel presented in **Table** **3**.

**Table 3:** Antibody panel used to stain leukocytes in mouse blood.

Antigen^)^	Fluorophore	Producer	Catalog #	Concentration [mg mL^−1^]	Amount/million cells [µg]
CD3e	PerCP	Biolegend	100325	0.2	0.5
CD19	FITC	Biolegend	152404	0.5	0.0625
CD11b	PE‐Cy7	Biolegend	101216	0.2	0.06
Ly6C	APC‐Cy7	Biolegend	128026	0.2	0.25
Ly6G	PE	Biolegend	127067	0.2	0.25

After 30 min of incubation at 4 °C, the cells were washed by adding 2 mL of FACS buffer and centrifuged at 500*g* for 5 min. Supernatants were removed and cells were finally resuspended in 100 µL of FACS buffer supplemented with DAPI 5 µg mL^−1^. Cells were then analyzed using a Cytoflex Flow cytometer using the gating strategy summarized in Figure S18 (Supporting Information).

### Therapeutic Efficacy of IL‐10 mRNA‐Loaded LNPs

The therapeutic potential of our LNPs formulation was assessed by loading them with mRNA encoding IL‐10.

The experiment included the following treatment groups: Healthy Untreated mice; untreated DSS colitis‐bearing mice; DSS colitis‐bearing mice treated with Luc mRNA‐loaded n‐LNPs; DSS colitis‐bearing mice treated with IL‐10 mRNA loaded 30‐n‐LNPs. LNPs equivalent to 20 µg of mRNA were injected retro‐orbitally in mice at days 3, 5, and 7 from the start of DSS treatment. Every treatment group included 5 animals to account for biological variability. The experiment was replicated three independent times overall.

The therapeutic efficacy of these treatments was assessed using multiple endpoints:

Body weight loss: for every mouse, body weight was monitored over 8 days from the start of DSS exposure. Treatment efficacy was assessed by its ability to prevent weight loss compared to DSS untreated mice.

Colon Length: prevention of colon shortening by different treatments was tested by sacrificing the mice on day 8 from the start of DSS exposure, harvesting the colons, and measuring them compared to DSS untreated mice.

Reduction of Local Pro‐Inflammatory Cytokines: expression of pro‐inflammatory cytokines was performed on the harvested colons as follows. Two 4mm diameter circular tissue biopsies (one from proximal and one from distal colons). Each part of the samples was then incubated in 400 µL of medium in a 24 wells plate overnight. The day after, the supernatant was recovered and centrifuged at 14000 rpm for 10 min to remove any cell debris. The final supernatant was decanted, transferred to Eppendorf tubes, and stored at −80 °C. ELISA assays were performed using ad hoc kits (R&D systems) to detect TNF‐α (Cat# DY410), IL‐12/IL‐13 p40 (Cat# DY2398) and IL‐6 (Cat# DY406). IL‐10 was also measured to validate the transfection efficiency of our LNPs in the colon of all mice groups (Cat# DY417). The results from each experiment were normalized by average levels of expression in the Healthy Untreated mice group.

Histology: The harvested colons were washed, cut longitudinally, rolled, and fixed in paraformaldehyde (Sigma–Aldrich, Cat # HT501128) to enable their inclusion in paraffin, sectioning, and hematoxylin/eosin staining. Tissue sectioning, staining, and imaging were performed by Histospek.

### Statistical Analysis

All data were presented as mean ± Standard Error (SEM). Statistical analyses for comparing two different groups were performed using a two‐tailed, paired Student's t‐test. Normality tests were performed to assume or exclude the data Gaussian distribution in the t‐tests. On the other hand, for experiments containing multiple groups, one‐way ANOVA with multiple comparison post hoc tests was employed instead. All these analyses were performed using Graph Pad Prism version 5.00 for Windows (Graph Pad Software, San Diego California USA, www.graphpad.com). *P* values below 0.05 were considered statistically significant. Significance intervals for *p* were designed as follows: ^*^for *p* < 0.05, ^**^for *p* < 0.01, ^***^for *p* < 0.001.

## Conflict of Interest

Listed in the manuscript under conflict of Interest (COI) only for Dan Peer. The rest of the authors have no COI.

## Supporting information



Supporting Information

## Data Availability

The data that support the findings of this study are available in the supplementary material of this article.
