# Supplementary material for: Lipid Nanoparticles With Fine‐Tuned Composition Show Enhanced Colon Targeting as a Platform for mRNA Therapeutics
Source: Adv Sci (Weinh). 2024 Nov 25;12(3):2408744. doi: 10.1002/advs.202408744 (PMC11744673; doi:10.1002/advs.202408744)
Supplement: Supplementary file 1 — Supporting Information [file ADVS-12-2408744-s001.docx]

Supporting Information

Lipid Nanoparticles with Fine-tuned Composition Show Enhance Colon Targeting as a Platform for mRNA Therapeutics

Riccardo Rampado ^1-4^, Somu Gonna Naidu^1-4^, Olga Karpov^1-4^, Meir Goldsmith^1-4^, Preeti Sharma^1-4^, Lior Stotsky^1-4^, Dor Breier^1-4^, Dan Peer^1-4, *^


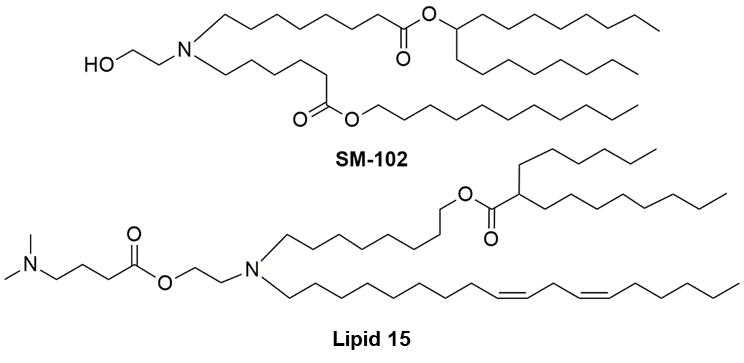


**Figure S1:** Chemical structures of the lipids employed in the study


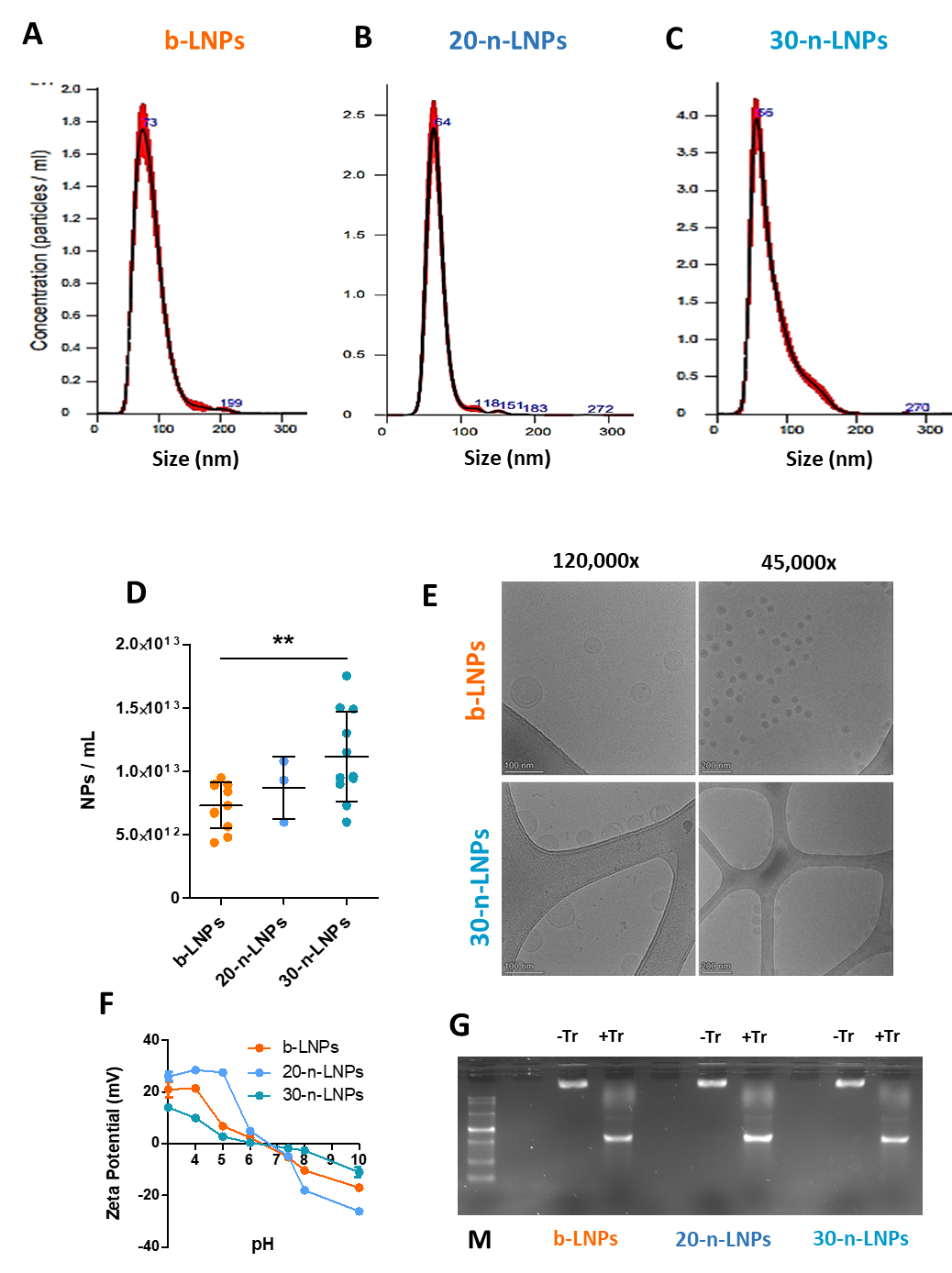


**Figure S2:** Representative size distribution of b-LNPs (A), 20-n-LNPs (B), and 30-n-LNPs (C), and their relative quantification of the NPs concentrations measured by NTA (n=10, D). E) Representative Cryo-EM images of b-LNPs and n-LNPs. F) Change of the NPs ζ across different pH of dispersant solution (n=2). G) representative agarose gel electrophoresis of NPs loaded with mLuc in absence (Tr-) or presence (Tr+) of Triton X-100. (All data are presented as average ± SEM; Statistical analysis was performed using a one-way ANOVA test, **: p<0.01).

**Figure S3:** Normalized viability of different cells lines after 72 hours incubation with increasing amounts of mLuc-b-LNPs or mLuc-n-LNPs measured by XTT assay (n=3, data are presented as average ± SEM)


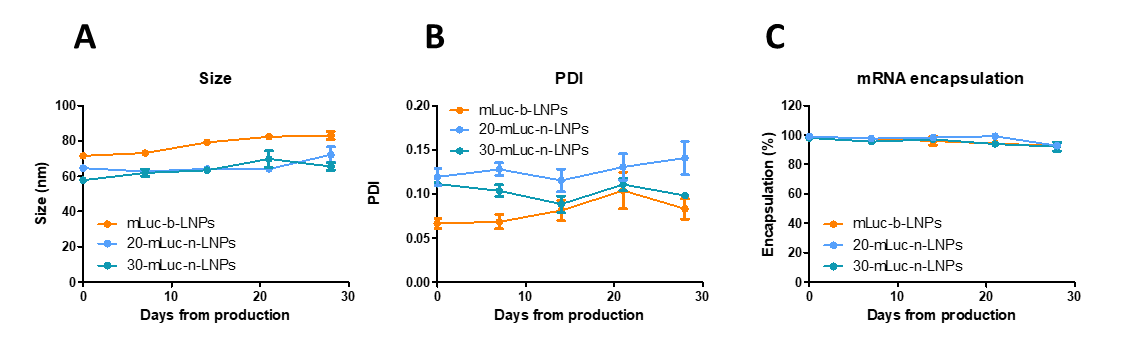


**Figure S4:** Measurement of LNPs size (A), PDI (B), mRNA encapsulation (C), over a period of 28 days of storage at 4°C (measurements performed on two independent formulations, data are presented as average ± SEM).


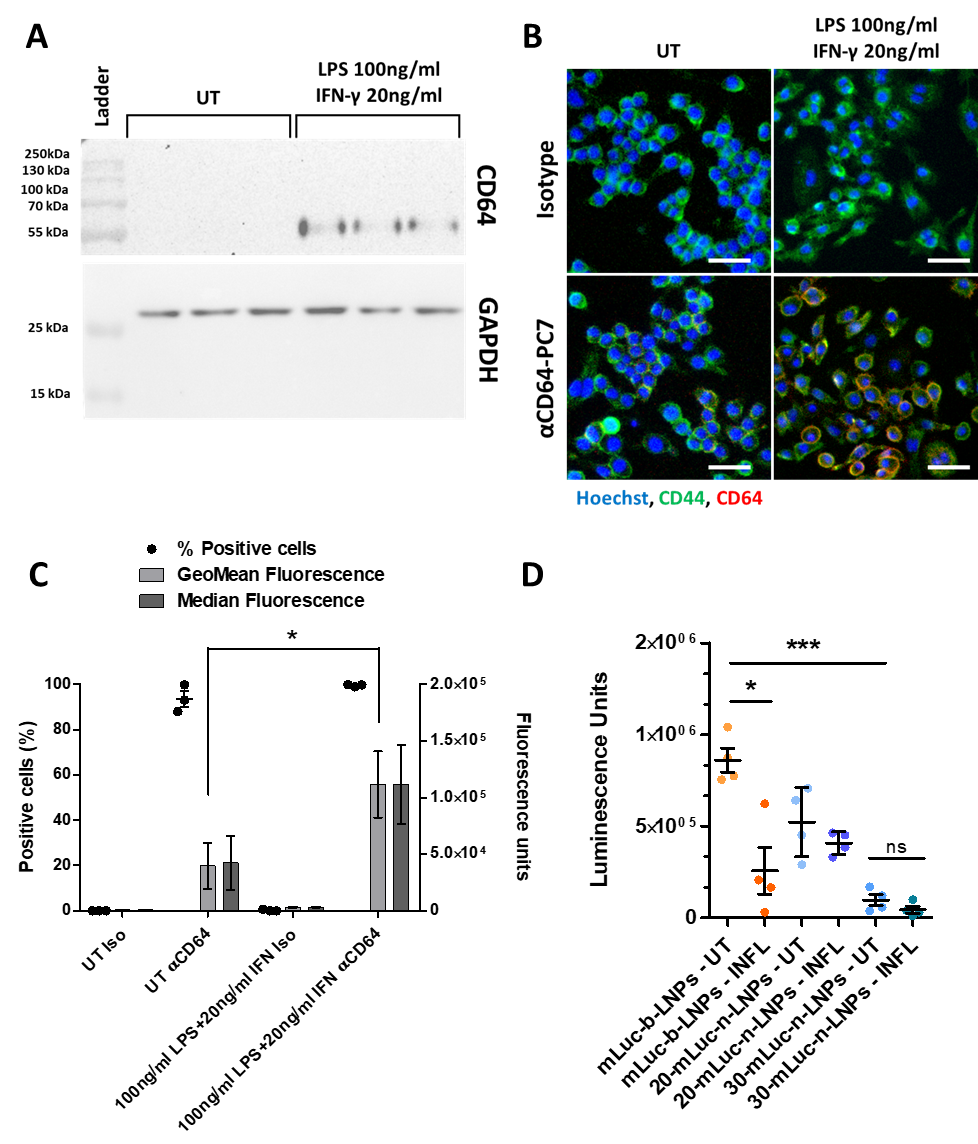


**Figure S5:** Assessment of CD64 expression measured in regular and inflamed RAW 264.7 cells using SDS-PAGE/western blot (A), fluorescence microscopy (B), and flow cytometry (C). D) measurement of Luc signal in regular (UT) or inflamed (INFL) RAW 264.7 cells after 24 of incubation with a dose of 0.25ug/mL of mRNA in b-LNPs, 20-n-LNPs, or 30-n-LNPs (n=4). All data are presented as average ± SEM. Statistical analysis was performed using a one-way ANOVA test, *: p,0.05, **: p<0.01, ***: p<0.001.

**Figure S6:** Luc expression measured in different cells lines after 24 hours incubation with 0.25 µg/mL of mLuc encapsulated in mLuc-b-LNPs, mLuc-20-n-LNPs or mLuc-30-n-LNPs (n=3). All data are presented as average ± SEM. Statistical analysis was performed using a one-way ANOVA test, *: p,0.05, **: p<0.01.


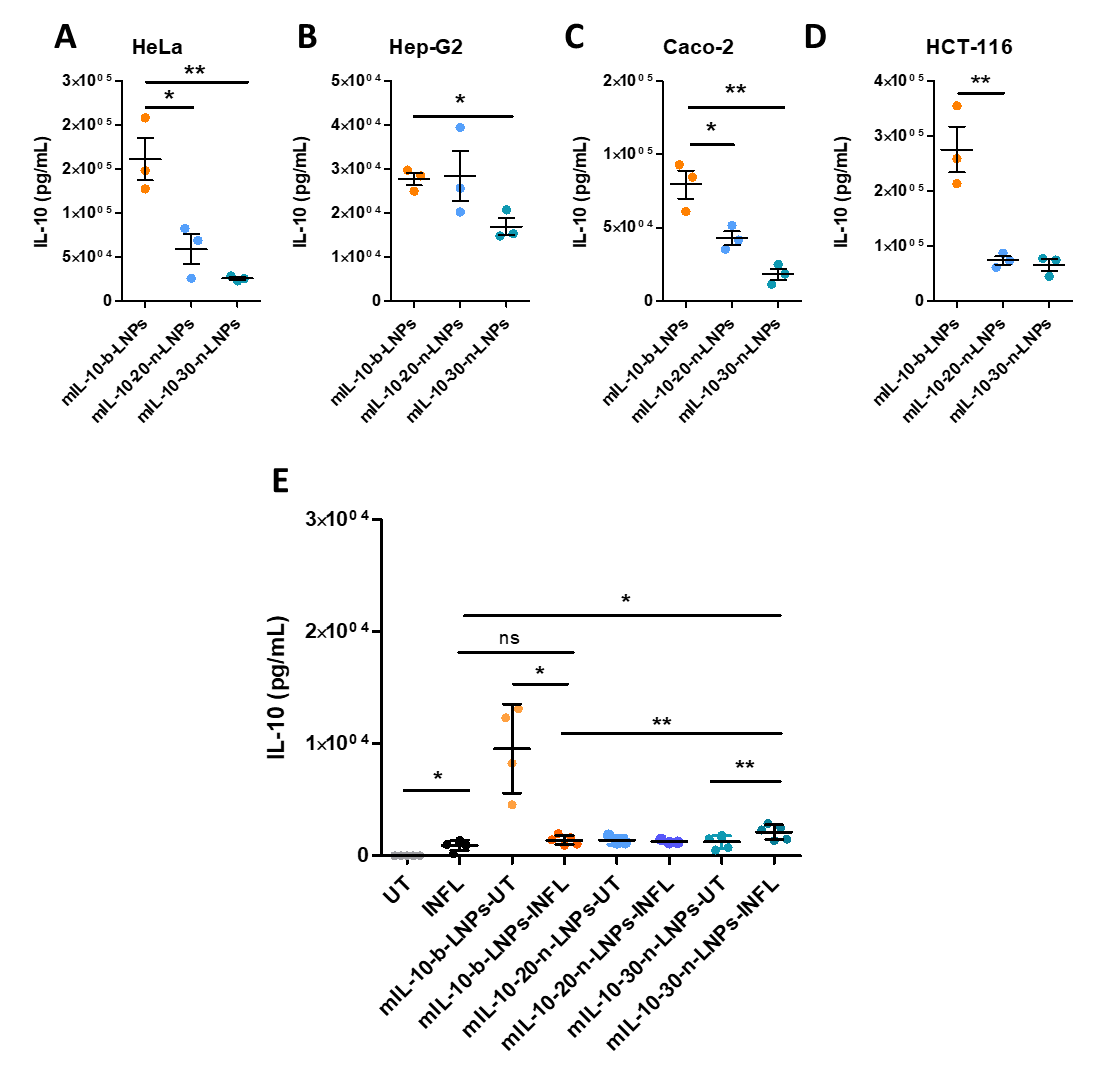


**Figure S7:** Assessment of IL-10 expression tested after 72 hour of incubation in HeLa (A), HepG2 (B), Caco2 (C), HCT-116 (D) and naïve (UT) or inflamed (INFL) RAW 254.7 cells (n=3 for A-D; n=4 for D). All data are presented as average ± SEM. Statistical analysis was performed using a one-way ANOVA test, *: p,0.05, **: p<0.01.


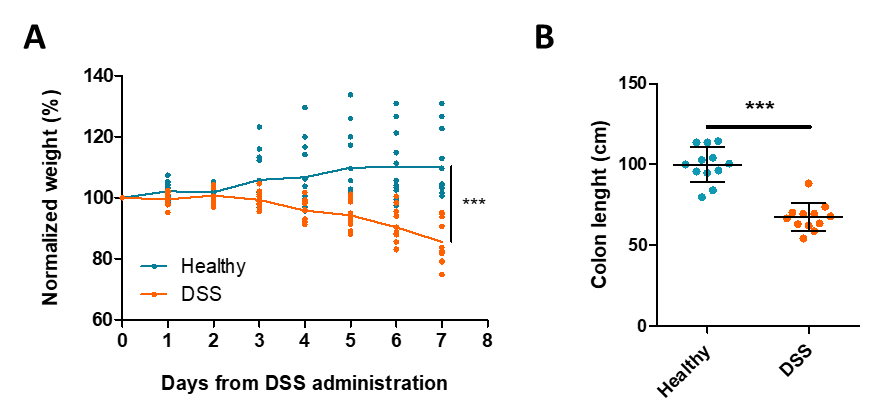


**Figure S8:** summarized data for the monitoring of the mice body weight (A) during DSS exposure over 7 days (12 mice included in each group, each mouse weight was normalized against its weight on day 0), and comparison of the colon lengths after mice sacrifice on day 7 (12 mice included in each group). Data are presented as average and single data points (A) or as average ± SEM (B). Statistical analysis was performed using a t test, ***: p<0.001.


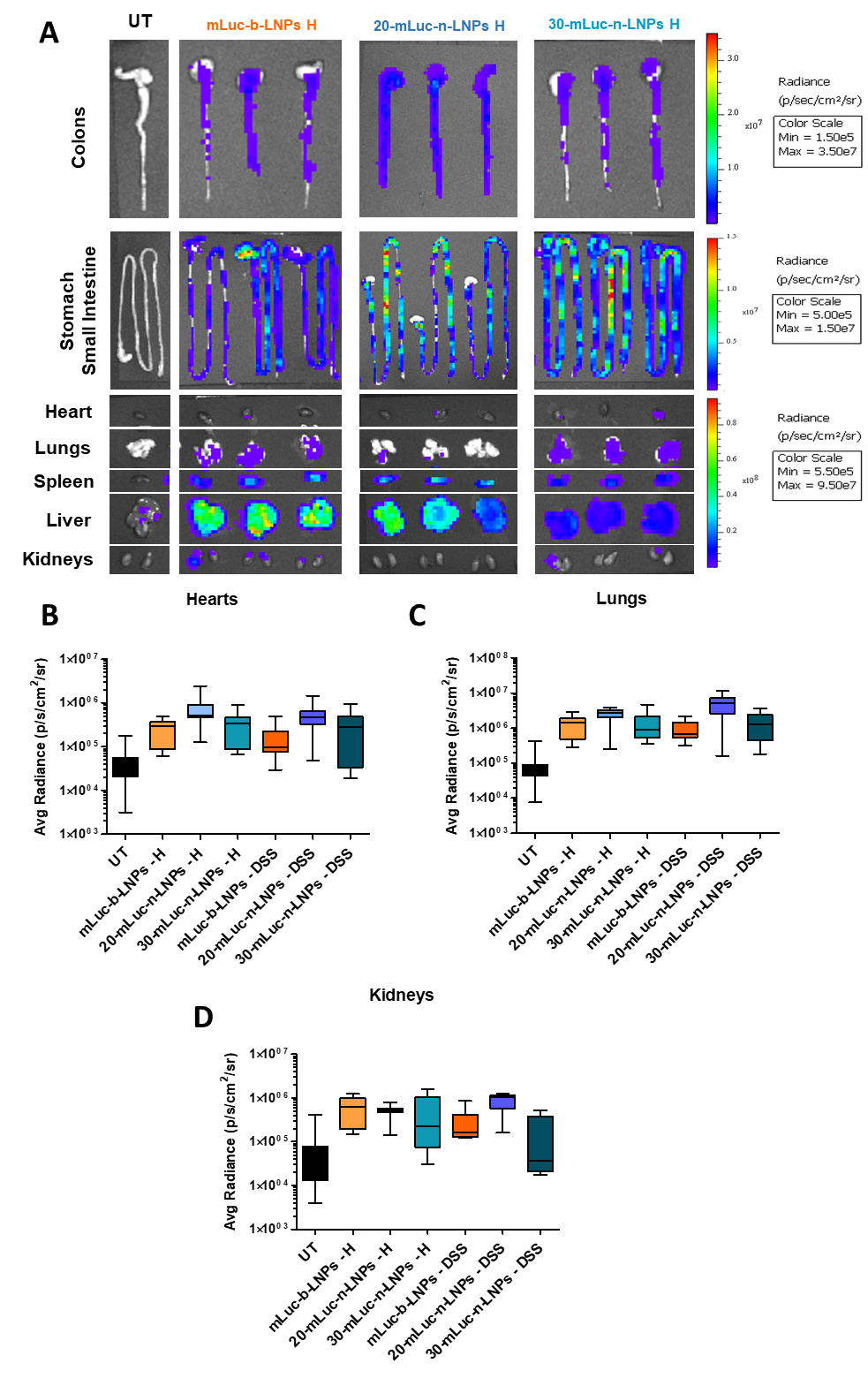


**Figure S9:** Representative IVIS image of healthy mice organs 6 hours from mLuc-LNPs injection (A) and their relative quantification for the hearts (B), lungs (C), and kidneys (D)(12 mice were included in each experimental group, data are presented as Box and Whiskers from minimum to maximum).


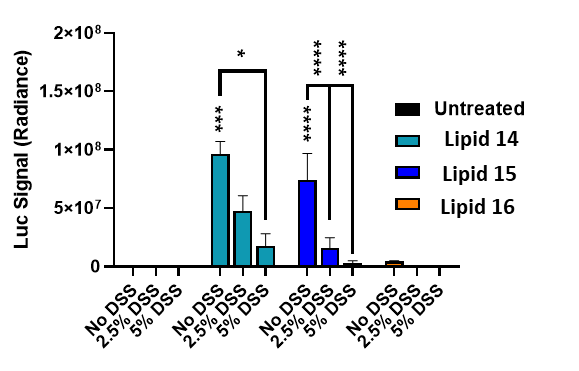


**Figure S10:** Assessment of the effect of DSS dose on the Luc expression in mice livers after administration with LNPs formulated with different ionizable lipids (10 mice were included in each experimental group). All data are presented as average ± SEM. Statistical analysis was performed using a one-way ANOVA test, *: p,0.05, **: p<0.01, ***: p<0.001, ****: p<0.0001.


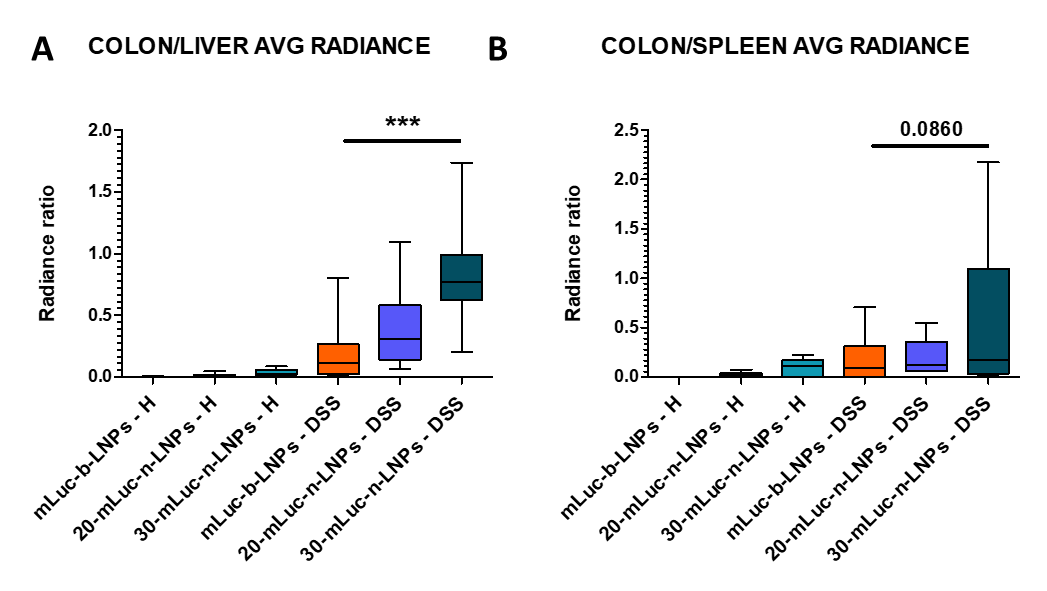


**Figure S11:** Calculation of the colon to liver (A) and colon to spleen (B) ratios between the Luc radiance signals measured in healthy or DSS colitic mice treated with mLuc-b-LNPS, mLuc-20-n0LNPs, or mLuc-30-n-LNPs (12 mice in each experimental group, data are presented as Box and Whiskers from minimum to maximum). Statistical analysis was performed using a one-way ANOVA test, ***: p<0.001.


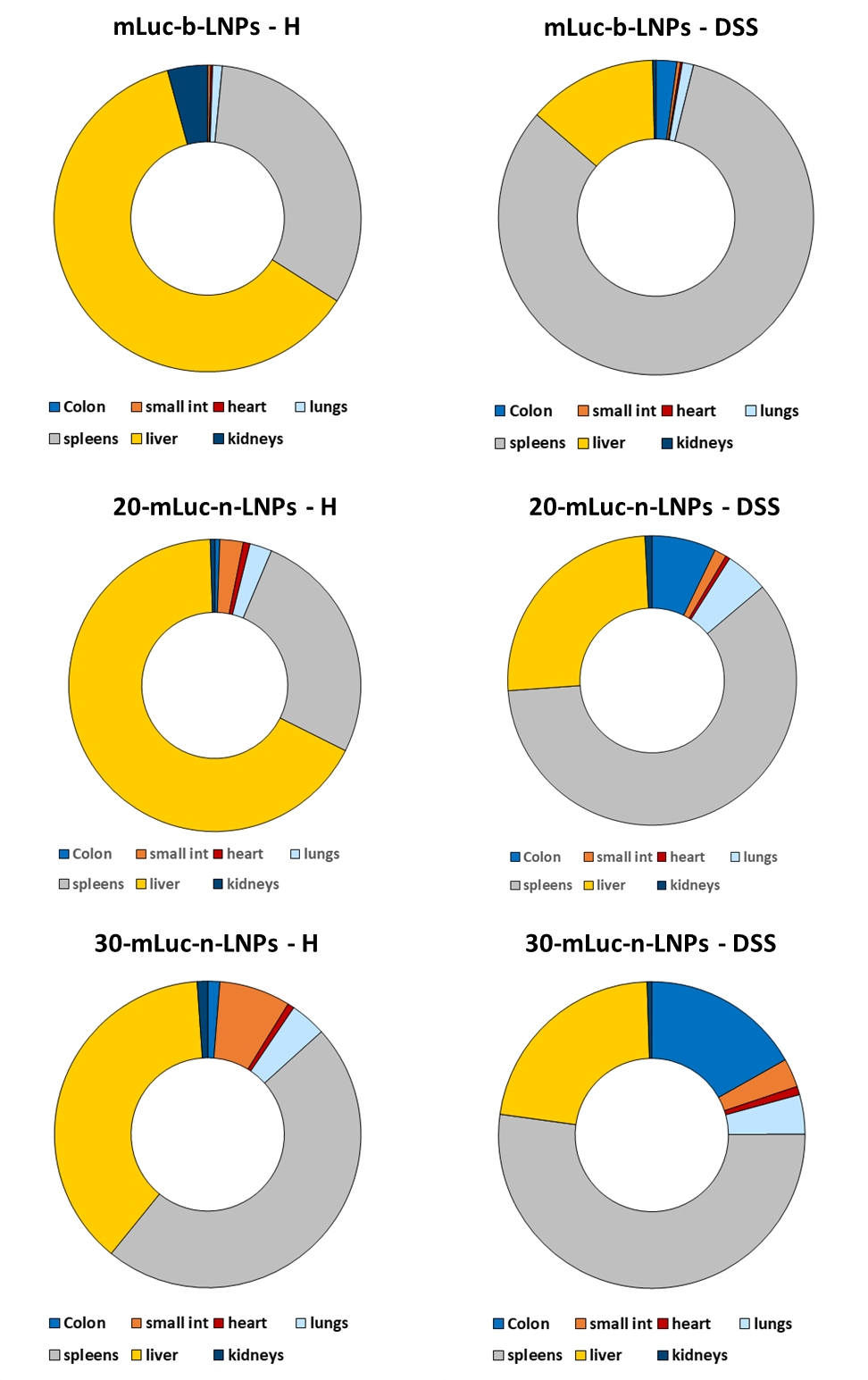


**Figure S12:** Representation as pie charts of the different fractions of Luc average radiance detected in healthy (H) or DSS colitis-bearing mice (DSS) after treatment with mLuc-b-LNPs or mLuc-n-LNPs (12 mice were included in each group). Pie chart areas prepresent the average Radiance for each organ.


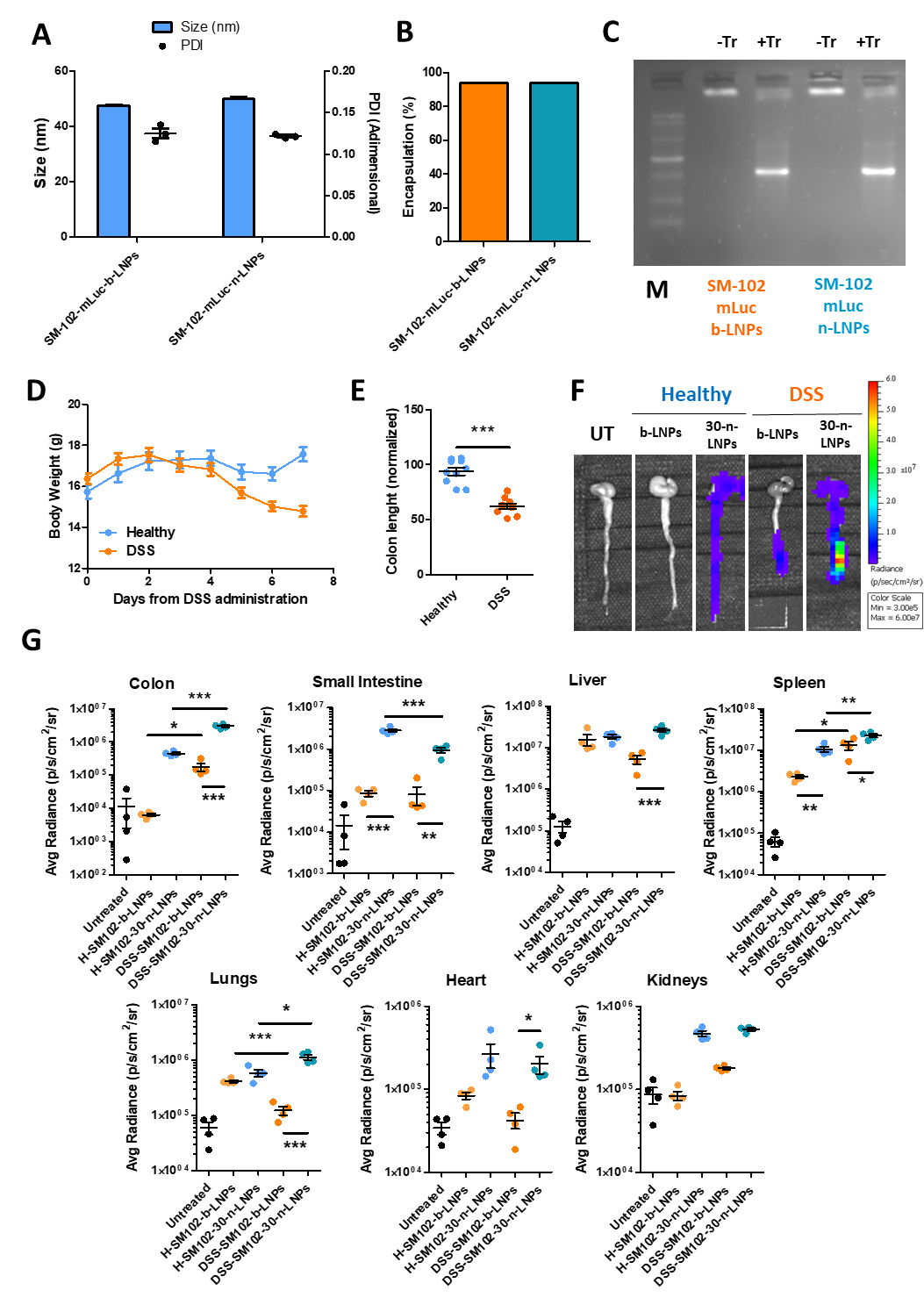


**Figure S13:** Characterization of b-LNPs and n-LNPs formulated with SM-102 as ionizable lipid for size (A), mRNA encapsulation (B) and agarose gel electrophoresis mRNA retention (C). Body weight (D) and measured colon length (E) of mice during exposure to DSS over a week to induce colitis. F) IVIS representative images and analysis of mLuc signal in main organs 6 hours after SM-102-LNPs injection (G, 4 mice were included in each group). Data are presented as averages ± SEM. Statistical analysis was performed using a one-way ANOVA test, *: p,0.05, **: p<0.01, ***: p<0.001, ****: p<0.0001.


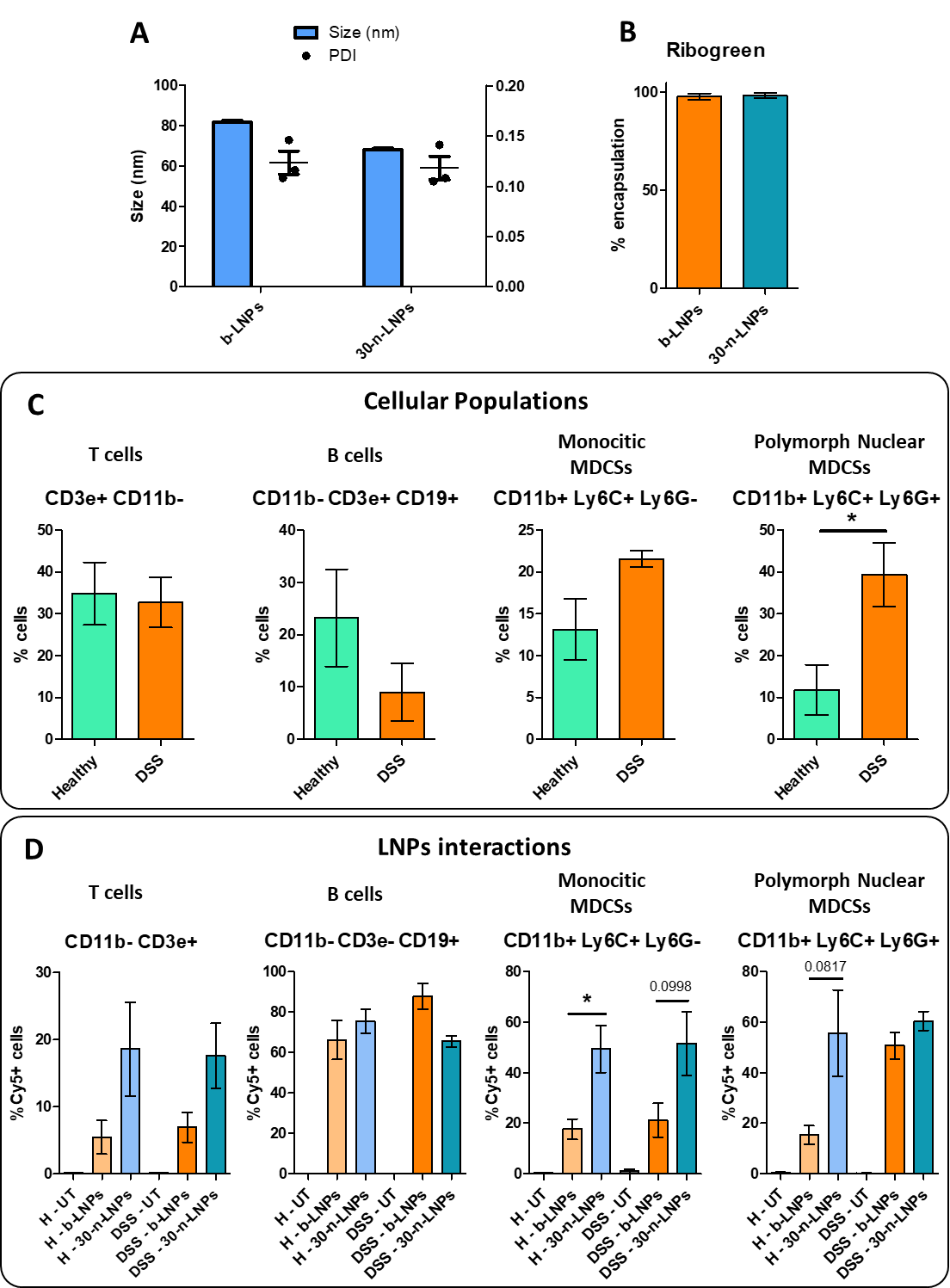


**Figure S14:** Assessment of the Size, PDI (A) and RNA encapsulation (B) of Cy5-loaded LNPs. C) Percentages of different leukocytes populations within the blood of healthy and DSS mice. D) Assessment of LNPs interactions with different immune cells subsets in healthy and DSS colitis-bearing mice (3 mice were included in each group). Data are presented as average ± SEM. Statistical analysis was performed using a t test, *: p,0.05.


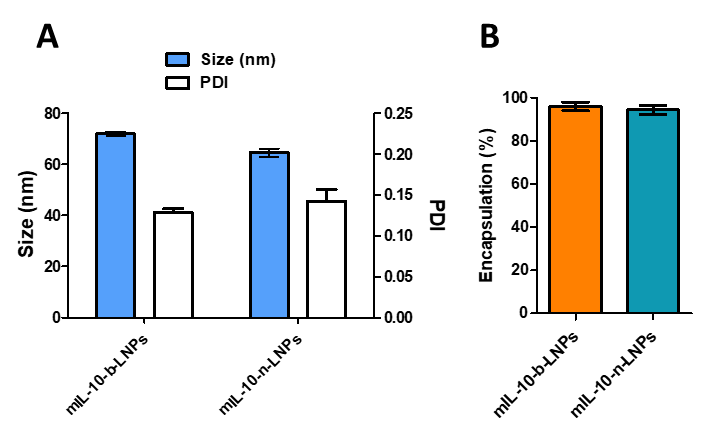


**Figure S15:** Characterization of mIL-10-b-LNPs and mIL-10-30-n-LNPs size (A), Encapsulation (B) (n=3). Data are presented as average ± SEM.

**Figure S16:** blood profile of C57BL/6J mice after 24 from the administration of LNPs corresponding to 20µg of mLuc mRNA. Data are presented as average ± SEM.


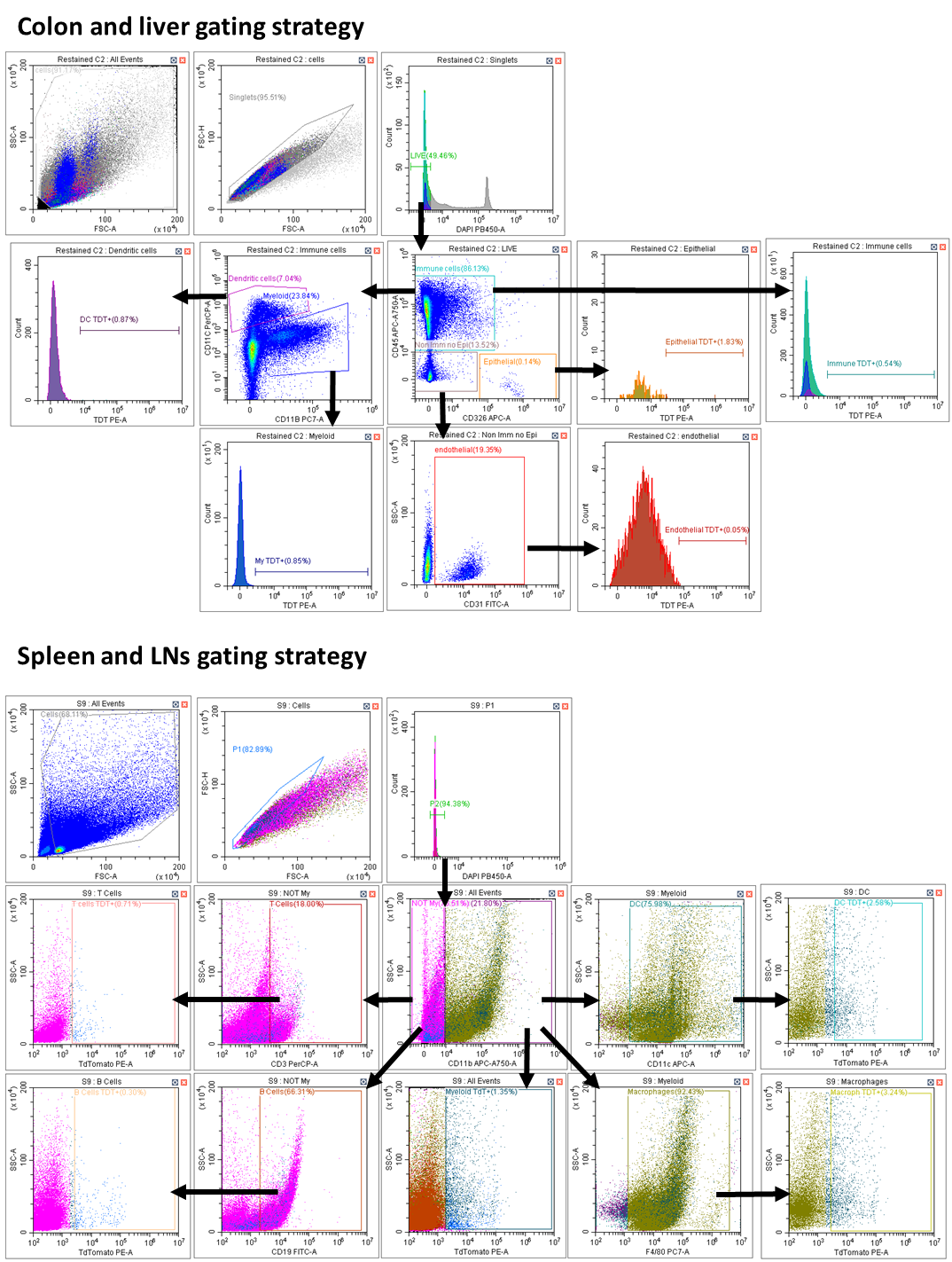


**Figure S17:** Gating strategies used for the flow cytometric analysis of Cre-tdTomato mouse cells extracted from the colons and liver (upper half) or spleen and mesenteric lymph nodes (lower half).


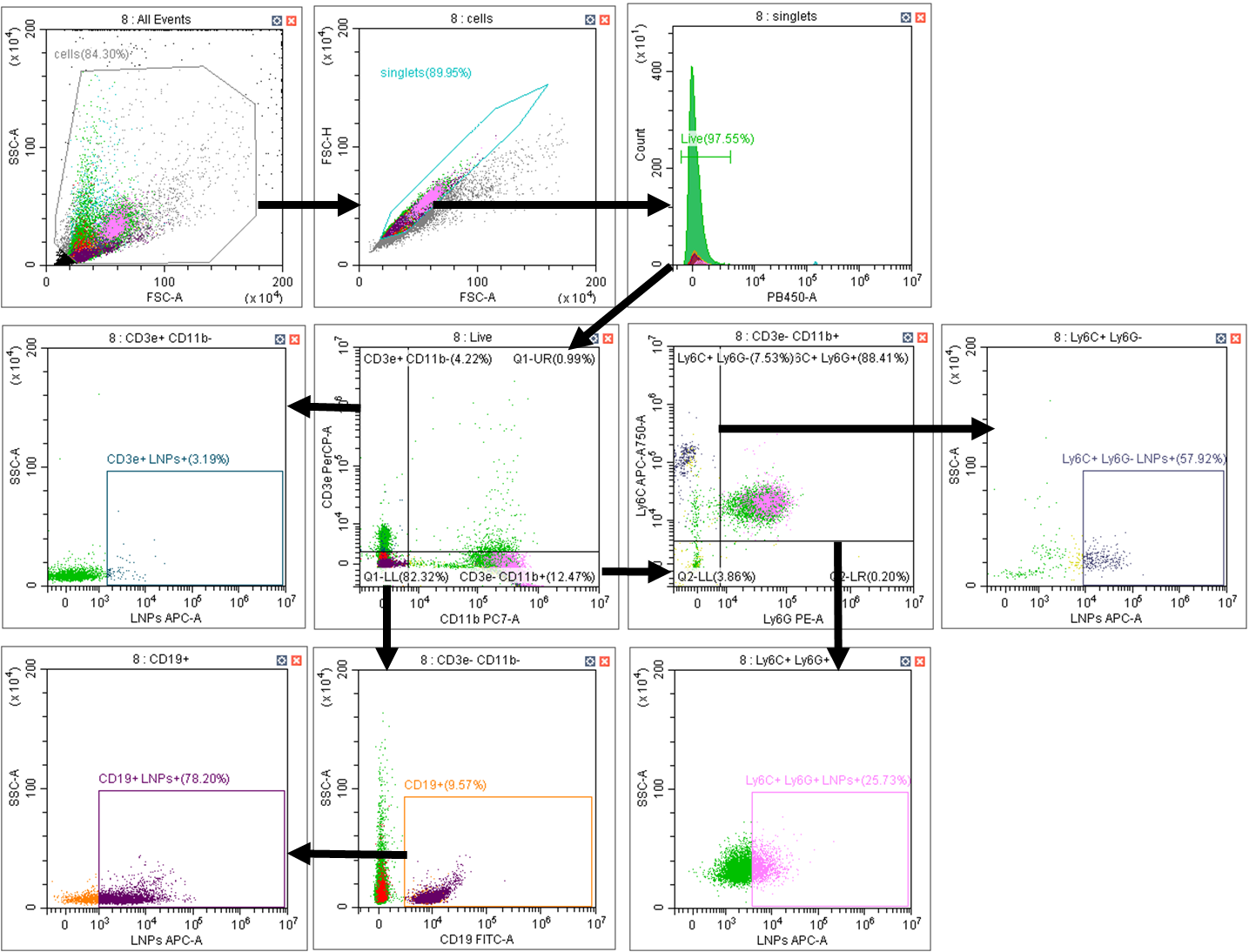


**Figure S18:** Gating strategy used for the flow cytometric analysis of leukocytes populations in mice blood and their interaction with LNPs.
